# Exploring Imaging Techniques for Detecting Tomato Spotted Wilt Virus (TSWV) Infection in Pepper (*Capsicum* spp.) Germplasms

**DOI:** 10.3390/plants13233447

**Published:** 2024-12-09

**Authors:** Eric Opoku Mensah, Hyeonseok Oh, Jiseon Song, Jeongho Baek

**Affiliations:** 1 Gene Engineering Division, National Institute of Agricultural Sciences, Rural Development Administration, Jeonju 54874, Republic of Korea; omedjin@gmail.com (E.O.M.); songmari@korea.kr (J.S.); 2 CSIR—Plant Genetic Resources Research Institute (CSIR-PGRRI), Bunso P.O. Box 7, Ghana; 3 National Agrobiodiversity Center, National Institute of Agricultural Sciences, Rural Development Administration, Jeonju 54874, Republic of Korea; zzjiy@korea.kr

**Keywords:** pepper, germplasm, TSWV, imaging, detection techniques

## Abstract

Due to the vulnerability of pepper (*Capsicum* spp.) and the virulence of tomato spotted wilt virus (TSWV), seasonal shortages and surges of prices are a challenge and thus threaten household income. Traditional bioassays for detecting TSWV, such as observation for symptoms and reverse transcription-PCR, are time-consuming, labor-intensive, and sometimes lack precision, highlighting the need for a faster and more reliable approach to plant disease assessment. Here, two imaging techniques—Red–Green–Blue (RGB) and hyperspectral imaging (using NDVI and wavelength intensities)—were compared with a bioassay method to study the incidence and severity of TSWV in different pepper accessions. The bioassay results gave TSWV an incidence from 0 to 100% among the accessions, while severity ranged from 0 to 5.68% based on RGB analysis. The normalized difference vegetative index (NDVI) scored from 0.21 to 0.23 for healthy spots on the leaf but from 0.14 to 0.19 for disease spots, depending on the severity of the damage. The peak reflectance of the disease spots on the leaves was identified in the visible light spectrum (430–470 nm) when spectral bands were studied in the broad spectrum (400.93–1004.5 nm). For the selected wavelength in the visible light spectrum, a high reflectance intensity of 340 to 430 was identified for disease areas, but between 270 and 290 for healthy leaves. RGB and hyperspectral imaging techniques can be recommended for precise and accurate detection and quantification of TSWV infection.

## 1. Introduction

Global demand for food is expected to increase by 50% by 2050. However, an estimated 10–40% loss in agricultural production can be attributed to plant diseases, including those caused by viruses [1,2]. Over 68–70 different types of viruses have been identified in pepper, and 20 are known to cause significant economic losses to production [3,4,5]. The volume of viral infection in pepper has increased over the past years, mainly because of the expansion of production, and increased trading that enhances the movement of the virus and the vectors across geographical boundaries [6]. Moreover, viral diseases such as cucumber mosaic virus (CMV), tobacco mosaic virus, (TMV), and tomato spotted wilt virus (TSWV) are common in pepper production worldwide [7,8,9]. Vectors transmitting viral diseases in pepper include aphids, thrips, and whiteflies. Also, these identified vectors easily develop resistance to insecticides [6], thus making the control of viral diseases a big challenge in the agricultural sector.

Tomato spotted wilt virus (TSWV), first described in 1919 in a tomato in Australia, remains one of the most prevalent viral diseases in modern times causing significant economic losses to pepper production [3,10,11]. TSWV is only second to tobacco mosaic virus (TMV) among the 10 most important plant viruses [12,13]. The virus raises interest scientifically and economically because it can self-replicate in both the vector and the host plant [14]. TSWV (genus *Orthotospovirus*, family *Tospoviridae*) is a negative and ambisense single-stranded RNA species and its pleomorphic particles are 80–120 nm diameter with two viral glycoproteins—GI and G2—as the surface projections [11,15]. TSWV and other tospoviruses (about 7 other tospoviruses) are related to diseases in over 1000 plant species and are the cause of losses of tens of millions of dollars, which is a global concern in crop production [16,17]. TSWV is transmitted by multiple thrips species, especially *Frankliniella occidentalis* (the order *Thysanoptera* and family *Thripidae*) [16]. Other trip species include *F. fusca*, *F. bispinosa*, *F. gemina*, *F. cephalica*, *Thrips tabaci*, and *T. schultzei* [18,19]. Temperature and suitable host are some of the seasonal factors that can affect the population of the thrips [18,20]. Temperature around 30 °C is conducive to female survival and the production of eggs in lower temperatures may be appropriate for the larval stages [21]. The thrip acquires the virus in the larval stage and transmits it in the adult stage [18]. First, the immature (first and second instar) thrip acquires TSWV from infected host plants, then the virus replicates as the vector matures, and then spreads the virus to other plants during the adult stage [18]. Some researchers indicate that the thrips are attracted to feeding on affected plants because the virus down-regulates some toxic alkaloids such as atropine and scopolamine in infected plants [22,23]. Thus, a mutualistic relationship exists, where the thrip transports the virus to a new host, where the virus makes colonization and feeding of host plants easy for the thrips by decreasing the plant toxins and defensive mechanisms. Some weed species have also been identified as natural hosts, and therefore as the reservoir of the pathogen during the inert planting seasons [11,24]. Symptoms of TSWV depend on the plant species as well as the age of the plants, strain of the virus, weather conditions, and nutritional status [25,26]. Common symptoms include chlorotic blotches, mottling of leaves, ringspots, and patterns of lines on the leaves and fruits [25,27].

TSWV has become one of the most problematic viruses in pepper production due to the ability of the thrip (*F. occidentalis*) to spread rapidly across geographical regions and its rapid resistance to insecticides [28]. The increasing volume and scope of spread of TSWV called for a search for intervention. A single dominant gene *Tsw*, identified in *Capsicum chinense,* was reported to confer resistance to TSWV [29]; however, the resistance level varies from one geographic region to another [30]. Moreover, new strains of TSWV arise easily in the presence of resistant accessions, and the ability of the virus to replicate in the vector may increase the genomic diversification of its population [31]. This was confirmed by Roggero [32], where TSWV caused a high proportion of damage to the *C. chinense* P1152225 resistant variety, as well as the susceptivity of a known resistant commercial pepper cultivar grown under a glasshouse. Breaking resistances in pepper calls for action on developing cultivars for broad-spectrum resistance to new or spontaneous strains.

Developing a resistant cultivar to reduce the effect of TSWV on pepper increases the need to identify quantitative trait loci (QTL), and automated phenotyping plays an important role in the identification process [33]. Furthermore, the prompt and timely identification of TSWV infection is important to reduce loss and safeguard profitability, while providing large and accurate data for decision-making [34]. Imaging technology has been utilized since the middle of the twentieth century [35] for plant disease analysis [36], due to the non-destructive advantage of collecting data on the same organism for a long experiment [37]. For example, the common bacterial blight by *Xanthomonas* spp. was better studied through RGB image analysis than the traditional visual assessments [38]. Also, spectral differences between the infected and healthy leaves of cucumber (*Cucumis stivus*) were detected in the light spectrum with accuracy, using the hyperspectral imaging method [39]. Confirming the precision of hyperspectral imaging in disease studies, Thomas et al. [40] indicated that reflectance measurements were better for early detection of powdery mildew infections when colonies could be detected even earlier than RGB imaging and other traditional methods. A report shows the generation of fluorescent images of plants with an automated disease lesion annotation of *Pseudomonas syringae* pv. *tomato-infected Arabidopsis* with precision [41]. Based on visual characteristics or spectral properties reflected by plant conditions, automated phenotyping can reduce human influence and errors in disease assessment [42,43]. On a further note, hyperspectral imaging analysis provides the unique spectral signature of objects and materials across an electromagnetic spectrum. The technique is used to detect plant diseases in time [44] and to provide the necessary managerial actions to reduce the spread of diseases [43]. Also, other optical techniques, machine learning, and imaging recognition techniques such as multispectral, RGB, thermography, chlorophyll fluorescence sensors, 3D scans, and Lidar have been used for disease detection and categorization [39,45,46,47,48,49]. Most importantly, the leaf color, shape, and texture have been used to study plant disease [44]. The precision in the detection of plant diseases can be attributed to the innovative methods of data segmentation and analysis that provide insight into plant-pathogen interactions [36]. These techniques are useful for breeding programs geared towards increased crop production and environmental sustainability [50,51].

Imaging techniques such as light microscopy, confocal laser scanning microscopy, and transmission electron microscopies are used to study plant virus-host interactions and a possible plant virus infection cycle [52]. Also, RGB, multi, and hyperspectral imaging approaches have been used in many dimensions to recognize visual symptoms of viruses, plant nutritional conditions, metabolic and biotic changes, and plant growth conditions [42]. These non-destructive methods of assessing viral diseases are important to enable the efficient and accurate study of disease incidence and severity of TSWV infection symptoms in pepper plants and other plant varieties. Imaging techniques can eventually replace the manual method of using scores for disease index, which is highly subjective and error-driven [53]. Not only the external assessments, but imaging methods such as thermal fluorescence techniques can also study physiological changes and other internal activities in the plant, thus making it a superior approach to studying plant diseases. Techniques such as the segmentation of disease areas on leaves, differences in reflectance between healthy and normal plants, and thermal radiations have been used through imaging to detect and quantify plant disease with precision [37,44,49,54]. The imaging approach can be a fast way to screen plant accessions for further breeding programs and provide time-point monitoring of plant diseases during production. In this research, we tested the hypothesis that an imaging approach can be used to quantify the severity of damage of TSWV to different accessions of pepper and the resistance levels to the virus. Specifically, we explored RGB and hyperspectral imaging approaches to study the inter- and intra-severity of damages of TSWV to pepper and the technique for identifying different symptoms of TSWV in pepper plants.

## 2. Results

### 2.1. Response of Pepper Germplasms to TSWV Infection

Based on the bioassay results, which involved disease symptom observation and virus detection in plants through RT-PCR after TSWV-YI inoculation, the fifteen accessions showed different percentages of disease incidence (Table 1). Eleven of the accessions, representing about 73%, had an incidence of TSWV between 80 and 100%. The *C. chinense* accessions mostly tested low for TSWV, while the *C. annuum*, *C. frutescens,* and the *C. baccatum* accessions had 80–100% disease incidence. IT103079, IT32436 (*C. annuum*), and IT164927 (*C. frutescens*) were the three accessions to have 100% incidence, while IT308738 and IT308753 (*C. chinense*) had no incidence after 4 weeks of TSWV inoculation. The IT284050 (*C. chinense*) and IT136625 (*Capsicum* sp.) accessions showed a moderate incidence of 40%, as the next accessions with the least incidence. In general, the incidence of TSWV differed among the accessions, the species, and the plant status classified.

### 2.2. RGB Imaging

We classified the accessions into low, moderate, and high disease severity, based on results from the RGB imaging analysis (Table 2). Accessions with disease severity percentages from 0.0 to 0.5% were low, between 0.5 and 1.0% were moderate, and above 1.0% were high. IT308738, IT308753, and IT218962 showed low severity status while IT158568 was the most affected, with 5.68% severity.

**Figure 1:**
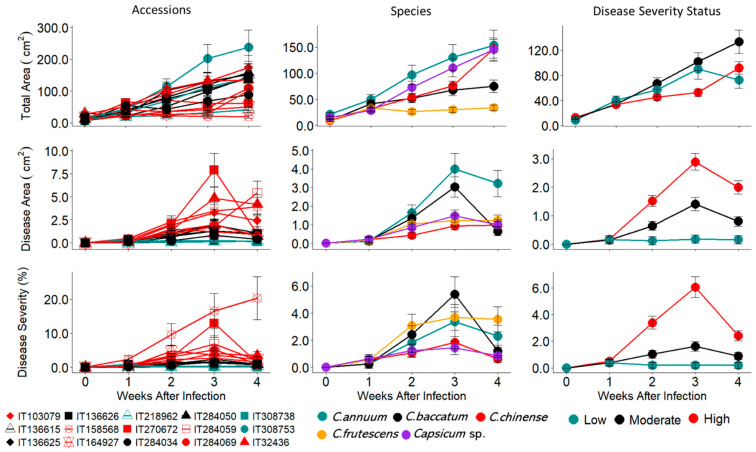
Line graph showing the progressive growth of plant accessions based on top-view area (cm^2^), disease area (cm^2^), and disease severity (%) of the accessions, species, and the disease severity status of the pepper infected with TSWV-YI. For the graph on accessions, the red lines represent accessions with high disease severity, the black lines represent accessions with moderate disease severity and the green lines represent accessions with low disease severity to TSWV-YI.

Accessions, species, and status of plant appearance to TSWV and time interactions were all significant (*p* = 0.000; Table A1) at the 0.05 probability level regarding plant canopy spread, disease area, and the TSWV infection percentage severity. Disease areas increased with time and canopy spread among the affected accessions. The IT308753 showed low severity and maximum growth with a maximum canopy spread of 238 cm^2^ at week four, compared with the other accessions of below 170 cm^2^ (Figure 1). The accessions IT158568, IT308738, IT218962, and IT164927 had a canopy spread below 50 cm^2^ after four weeks of infection. In general, TSWV severity on the pepper accessions peaked at week three after infection, causing more damage and loss of leaves. The percentage severity of IT158568 was the highest, while IT218962, IT308738, and IT308753 were the lowest.

**Figure 2:**
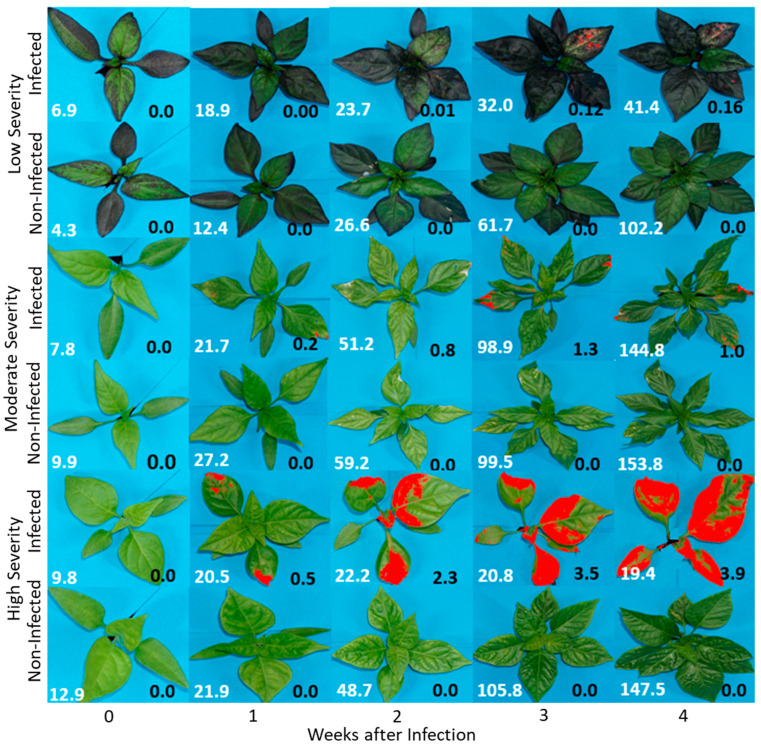
RGB images showing infection trends among *low*, *moderate*, and *high* disease severity to TSWV-YI infection of three selected accessions (IT218962—low severity, IT136625—moderate severity, and IT158568—high severity). Infected—plants inoculated with the TSWV; non-infected—the non-inoculated plants (control). The values in white are the average total areas (in cm^2^) of the five plants per accession, measured by the top-view camera at a distance of 0.6 m from the plants, while the values in black are the average infected parts (in cm^2^) of the five plants per accession, based on the image segmentation.

The *C. baccatum* species showed the highest disease severity damage of 5.4%, followed by the *C. frutescens* and *C. annuum* with 3.5% and 3.4%, respectively. The *Capsicum* sp. had the lowest severity of 1.4%, while *C. chinense* had 1.8% at week three, the peak severity of TSWV infection. We compared three selected accessions (IT218962, IT136625, and IT158568) as special cases (Figure 2). For the IT218962 accession, the disease area increased slightly from 0.0 to 0.16 cm^2^ four weeks after infection, making it classifiable as low severity accession, but canopy spread was from 6.9 to 41.4 cm^2,^ compared with the non-infected plant from 4.3 to 102.2 cm^2^. The trend shows a growth rate of 8.3 cm^2^ week^−1^ for the infected plants, against the normal plant of 24.8 cm^2^ week^−1^. For the moderate severity accession (IT136625), the growth rate was 34.2 cm^2^ week^−1^, similar to 36.0 cm^2^ week^−1^ of the non-infected. On the other hand, the high severity accession (IT158568) showed a declining canopy spread of 1.3 cm^2^ week^−1^ after two weeks of infection, due to loss of leaves, compared with the normal plant of 33.2 cm^2^ week^−1^. The three scenarios show the different responses of the pepper accessions to TSWV from the RGB image analysis.

### 2.3. Hyperspectral Analysis

**Figure 3:**
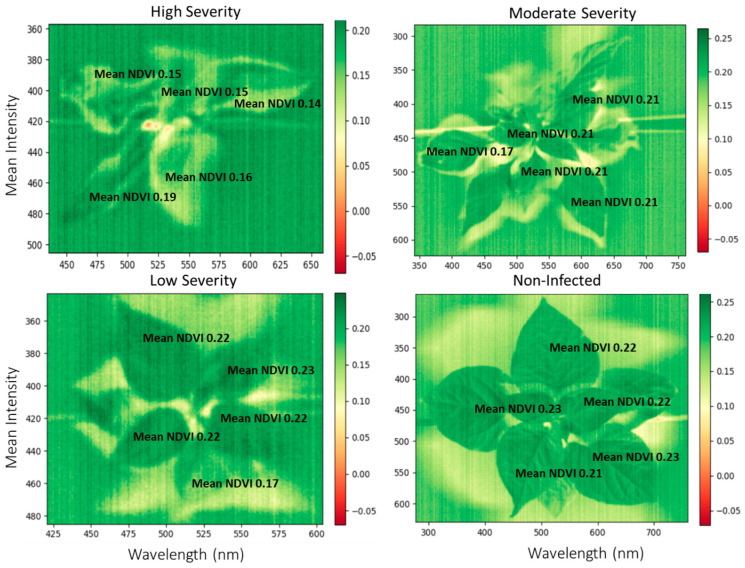
Hyperspectral analysis—hyperspectral images showing normalized difference vegetation index (NDVI) of selected accessions (IT218962—low severity, IT136625—moderate severity, IT158568—high severity, and IT284034—non-infected) based on TSWV symptoms assessments.

The normalized difference vegetative index (NDVI) graph displays the greenness of the pepper leaves based on the levels of infection by the TSWV (Figure 3). The parts of the leaves with infection had a low index of around 0.14–0.19, while the green leaves without infection ranged from 0.21 to 0.23. The high disease severity accession had most of the leaves indexed between 0.14 and 0.16. In contrast, the moderate disease severity accession showed an index of around 0.21, but the low disease severity accession had a parallel index with the control.

**Figure 4:**
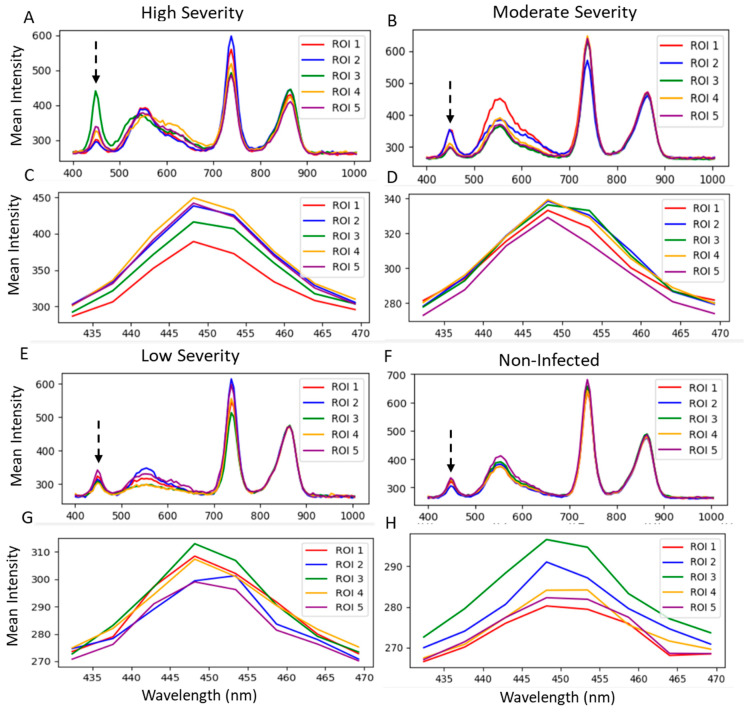
Hyperspectral reflectance of five spots on the leaf surfaces of high, moderate, and low severities, and non-infected plants. (**A**,**B**,**E**,**F**) from 400.93 to 1004.5 nm and (**C**,**D**,**G**,**H**) from 430 to 470 nm wavelengths on the electromagnetic spectrum. The bands indicate the number of spots selected on the plants. The broken black arrows show the band spot at which the infected areas were separated from the normal areas, and where symptoms of TSWV were assumed to be detected.

The spectral analysis shows TSWV symptoms in the wavelength between 430 and 470 nm, mostly in the visible light spectrum (Figure 4). Accessions with severely damaged leaf samples showed differences compared with non-infected leaf parts. The mean intensity of severely damaged leaf parts could reach 430, while the least damaged parts had low mean intensities of around 290.

### 2.4. Common TSWV Symptoms of Pepper

**Figure 5:**
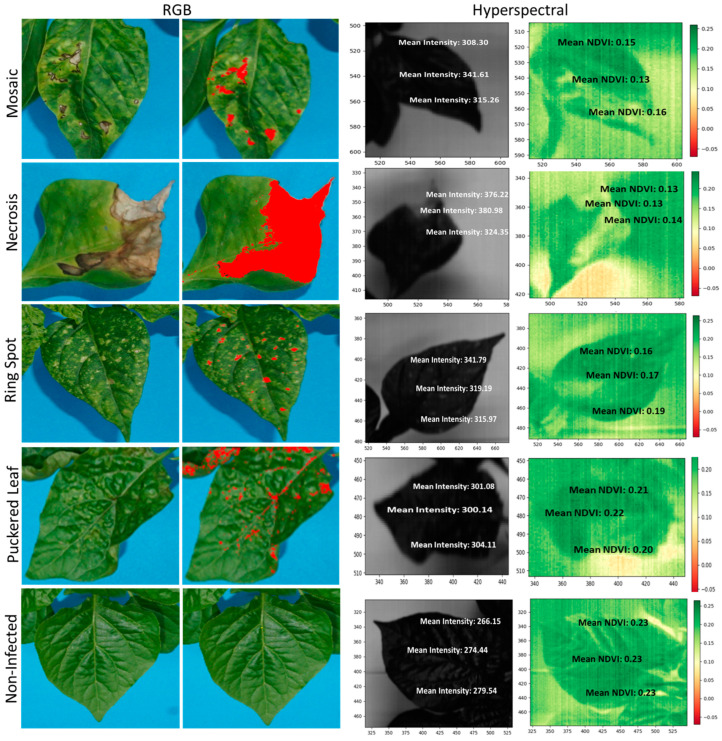
RGB and hyperspectral imaging for TSWV-YI common symptoms in pepper germplasm—mosaic, necrosis, ring spot, puckered leaf, and non-infected plant. NDVI—normalized difference vegetation index.

The assessment of common symptoms of TSWV in pepper germplasms shows the effectiveness of imaging techniques in quantifying the levels of damage by the disease (Figure 5). The original images of Figure 5 can be found in the Supplementary Materials.

Different symptoms showed similar patterns of quantifications in terms of RGB assessment, band intensities, and NDVI values. Based on the selected symptoms, the disease spots on the leaves reached high-band intensities ranging from 315.26 to 380.98, but as low as 266.15 for healthy spots. The results were in the opposite direction for the NDVI values. The disease spots on the leaves, excluding puckered leaves, had low ratios ranging between 0.13 and 0.19 depending on the intensity of damage, but the healthy spots had ratios as high as 0.23. Thus, high-band intensities or low NDVI values show high levels of damage across the various symptoms selected. The RGB assessment was based on the leaf area infected compared with the total leaf area across the different symptoms, and it would need further calculations to determine the severity of the symptom. Puckered leaf symptoms were a challenge, as the greenness of the infected parts was similar to the healthy parts of the leaves, compared with the necrotic symptoms that showed a sharp contrast. Using RGB and NDVI imaging techniques showed similar challenges.

## 3. Discussion

The use of bioassay, RGB, and hyperspectral imaging for disease validation provides concrete information on TSWV disease incidence and the severity of different pepper accessions. This research indicates that imaging, including RGB and hyperspectral, can replace the manual method of assessing plant diseases. Further research and development of imaging techniques can help precisely and accurately measure the incidence and severity of plant viral diseases such as TSWV and other important plant viruses.

Based on the bioassay results, the fifteen pepper accessions showed different responses. Few accessions showed low incidence with values between 0 and 40%. We provide two main reasons: either the TSWV infection was unsuccessful, or the plants had inherent resistances; therefore, the newly developing leaves sampled for the RT-PCR test did not show the virus. For example, IT308738 and IT308753, which may exhibit some levels of resistance to TSWV, had a zero percent disease incidence and a low severity percentage based on the bioassay results and the RGB imaging. Plants have natural ways to develop resistance to viral diseases. These include but are not limited to, antiviral mechanisms (including resistance to viral movement to new parts), translational repression, RNA silencing, innate immunity, and autophagy-mediated degradation [55,56]. After entry into their host, virulence strategies are activated by the viruses [57]. The virulence strategies include using the host DNA to replicate their genomes and to establish infection [58]. Viruses continue to synthesize new coats for encapsulation and then produce other virion particles. Virion particles move from cell to cell and from host to host with the help of vectors or other mechanisms [55]. Plants employ different mechanisms to either reduce cell-to-cell movement or deactivate the synthesis of new protein coats on the virus [59]. A low incidence and severity of TSWV in germplasms, including, IT308738, IT308753, IT284050, and IT136625, provide information on some levels of resistant mechanisms against the virus. However, further research is needed on such germplasms to confirm these claims. For now, the *Tsw* single dominant gene in pepper has been used by breeders to produce resistant accessions to TSWV [58] but recent studies have reported new strains of the virus breaking the resistance in pepper in the Republic of Korea [23,60]. Nevertheless, further trait studies and cultivar selection may uncover other traits that may provide broad-spectrum resistance to TSWV. The other 11 germplasms showed a high incidence of TSWV, with between 80 and 100% of the plants testing positive. However, some accessions had a high incidence but low severity. For example, IT218962 and IT136626 had an 80% incidence but 0.14% and 0.72% severity, respectively. These germplasms may either have genes to fight the virus to reduce severe damage or may localize the spread of the virus in tissues to limit their impact on the plant’s health [55,61]. Thus, the defense mechanism of the plants can result in high incidence but low severity among some pepper germplasms with TSWV. Germplasms with high incidence and severity, such as IT158568, IT164927, and IT270672, may have weak hormonal activities allowing the virus to take advantage of it [62], or other possible mechanisms. The 11 accessions are about 73% of the total pepper genotypes used for the trial, reflecting the number of genotypes on the farmers’ field that could be affected by TSWV and thereby pose a global challenge to pepper production.

We assessed the canopy spread, disease area, and percentage severity of the diseases using a top-view RGB camera. Pepper accessions such as IT308753 had active growth rates and showed mild symptoms from the infections of the virus. IT158568, IT270672, and IT103079 showed severe symptoms, possibly due to their leaf morphology (broad leaves) and the weak resistance to TSWV. Severity was high during the third week of infection, resulting in a loss of leaves and a decreased canopy spread during the fourth week. Reduction in canopy spread may be the plants’ mechanism to reduce surface area for infection and other physiological responses. Plants normally change their root and leaf morphology to overcome some of the stresses posed [63,64], but the changes can also cause detrimental damage.

The *Capsicum annuum* and *Capsicum* sp. showed more vigorous growth than the other species; however, symptoms of the viral infection tended to be pronounced among the *C. annuum* species, though the percentage of disease severity was higher among the *C. baccatum* species. The observation might result from the higher total area to the disease area ratio among the *C. annuum* species. Unlike *C. baccatum, C. annuum* might have a larger surface area than disease area, to help with physiological activities. Another confirmed defense mechanism of *C. annuum* is to restrict the movement of the virions to the upper leaves [65], though this mechanism has collapsed in other viral research [66,67]. Contrary to this work, *C. baccatum* is reported to demonstrate a promising source of resistance to many pepper diseases [68]. In this research, only two accessions of *C. baccatum* were used, and the few numbers might have contributed to our observation. On the other hand, *C. chinense* dominated the selection but generally showed lower disease area and severity. Previous studies have shown that many *C. chinense* collected from around the world exhibited low disease severity in bioassay [68], and were found to possess the *tsw* gene through marker analysis [69]. Compared to these findings, it is assumed that the *C. chinense* with low severity in our bioassay may also exhibit resistance to the *tsw* resistance-breaking TSWV variant (TSWV-YI) used in this experiment.

Comparing the growth pattern of three selected germplasms, the low severity (IT218962) accession showed subtle symptoms of TSWV, but the bioassay confirmed the presence of the virus. Based on the growth rate, the main symptom of TSWV for IT218962 was stuntedness, which was difficult to identify using only the disease segmentation process in RGB imaging. Automated disease analysis of the germplasms (noted by high anthocyanin content on the leaves) may need to focus on area growth, instead of the other symptoms like leaf necrosis identified on the other germplasms. Though the IT136625 accession had some more symptoms than the IT218962, it was strong and its growth was faster. On the other hand, the IT158568 accession showed weak responses with time, losing several leaves. Different accessions of pepper have different resistance mechanisms to viral diseases. Recessive and dominant genes mediate the resistance mechanisms [70,71]. While the recessive resistances may result from a mutation that causes incompatible interaction between the plant and the virus, the dominant resistances come from a compatible interaction between a viral effector and the plant R proteins, leading to the weakening activities of the virus [66]. The recessive genes are common and durable, and provide a more broad-spectrum resistance than the dominant genes [72]. The resistance mechanisms influence plants’ ability to fight foreign protein particles, and they differ among *Capsicum* species [65,73].

To monitor the spread of TSWV and other plant diseases, a remote and proximal sensing approach is paramount to ensure early intervention. The normalized difference vegetative index (NDVI) could be one of the remote sensing approaches to validate disease damage by TSWV. The technique has been used to monitor plant biomass and nutrient content status [74] and would be a novel way to study plant diseases. Since the NDVI measures the greenness of the leaves [75], it could be employed to study the severity of damage to the leaves. For the selected examples, the NDVI values of the disease area were low, around 0.14–0.19, while the healthy green parts were high, around 0.23. A similar result is reported by [59], who identified decreased NDVI values for the severity of *Septoria tritici* in winter wheat. Unlike manual assessment, NDVI is fast and can quantify the severity of the damage at a spot and provide a standard value for further analysis. The NDVI technique may have to be used with bioassay to confirm the presence of the virus, since other symptoms similar to chlorotic or necrotic leaves may also show the same results. Analyzing the activity of TSWV under 400.93 to 1004.5 nm band size showed similar waveband patterns across the accessions and TSWV infections. However, the peak in the intensities differed between the infected spots and healthy spots in the visible light spectrum [76]. Berdugo et al. [39] showed peak differences in the infected and healthy parts of cucumber plants in the visible light range, confirming our results. A validation of the precision of hyperspectral reflectance in plant diseases detection is also reported by Thomas et al. [40] in powdery mildew and barley research, Gu et al. [77] in TSWV and tobacco research, and Wang et al. [78] in TSWV and hyperspectral modeling projects. In another development, RGB and hyperspectral image analysis techniques have proven to be novel approaches for fungal diseases, compared with the traditional methods [79]. These reports indicate the possible application of imaging techniques in detecting other plant diseases and replacing the already-known traditional methods. In the 430 to 470 nm range in the visible light spectrum and in particular, the blue/blue-green edge [80], the sensitivity of the TSWV was pronounced. At this spectral band range, wide differences in band intensity in the normal and infected spots were assumed to show the virulence activity of the TSWV on the different accessions of pepper. We confirmed this by comparing it with non-infected leaves, and the differences were visible. These characteristic changes have also been observed in soybeans from yellow mosaic disease [81] and potatoes from potato yellow vein virus [82]. Earlier reports indicate the difference in leaf pigmentation being the important factor for discrimination, as chlorophyll a and b dominate absorption in the visible light region [80]. With further dimensionality reduction in the band range from 430 to 470 nm, the infected parts of the plant showed high intensity, while the non-infected leaves showed low intensity. This was across all the accessions studied. This primary validation exercise could be a fast way to identify and study the severity of viral activities in pepper plants and other food crops.

Common symptoms identified on the fifteen pepper accessions included severe mosaic, puckered leaves, ring spots, stem and leaf necrosis, and stunted growth. Symptoms such as necrosis, ring spots, and mosaics were easily segmented using the RGB. The imaging technique has also been used to precisely study pustule number, pustule size, leaf area, and pustule coverage in pea leaflets [44]. However, puckered leaf symptoms were challenging, since the leaf area was difficult to calculate due to curling and the almost green pigmentation resembling a normal leaf in the RGB view. Imaging approaches use contrast mechanisms between the disease area and the healthy leaf background [44]; therefore, symptoms that do not have such sharp contrast are difficult to segment and quantify. Using the NDVI and hyperspectral imaging techniques, precise disease spot identification on the leaf is important to analyze the severity of the disease. When the ringspots are tiny or with puckered leaves, magnification of the spot is important to get the exact point for measurement. In general, both RGB and hyperspectral analysis can effectively study the severity of damage by TSWV.

The spread of TSWV and the mass destruction of crop varieties is a global challenge. Bioassay is a surety to identify the presence of the virus on the plant. However, the process is destructive, costly, and time-consuming. RGB and hyperspectral imaging techniques would be appropriate to identify plant diseases apart from the bioassay. Hyperspectral imaging has been used intensively in various research fields to study the spectral features of plant diseases and is now advancing to the spatial spectrum [83], paving the way for more research in field studies of plant diseases. Mostly, the severity of infection could be detected with precision at different plant growth stages while in the field, as well as the complex interactive factors in the plant’s ecosystem. Since TSWV does not have a cure, advanced study of the imaging technique can be used in field trials to monitor the presence and the population of the vector (*Frankliniella occidentalis*) on the field. The prompt monitoring of the vector can provide information on its cyclic activities, and a timely intervention to control the spread of the virus from one plant population to another. Nevertheless, other conventional approaches such as farm hygiene, removal of infected plants, bottom pruning, crop rotation, mulching, the use of biological control of vectors, and plant-resistant accessions can be used to reduce viral infections. Further development of a user-friendly app for TSWV and other viral disease identification techniques through imaging could reduce production losses and increase food security.

## 4. Materials and Methods

The research was conducted at the National Agrobiodiversity Center (NAC) under the Rural Development Administration (RDA), Jeonju, Republic of Korea, between June and October 2024.

### 4.1. Plant Materials

Fifteen accessions of pepper with different levels of responses to tomato spotted wilt virus (TSWV) were obtained from the gene bank of the NAC (Table 1). These accessions represent five species (*Capsicum annuum*: 2, *C. baccatum*: 2, *C. chinense*: 5, *C. frutescens*: 3, and *Capsicum* sp.: 3) sourced from various countries (Table 1). Seeds of the obtained pepper were nursed into a 50-cell tray with a seed per cell and kept in the greenhouse of the NAC research field in June. Two weeks after sowing, six healthy seedlings per accession were selected from the germination trays and transplanted into 10 cm-wide pots filled with commercial horticultural soil (Hanarum, Shinsung Mineral Co., Ltd., Goesan, Republic of Korea) and grown using standard agronomic practices according to RDA cultivation methods from June to August. The greenhouse was maintained at 28 ± 5 °C in the growing season and kept free from insects.

### 4.2. Bioassay for TSWV Infection

A week after transplanting and at the three to four true-leaf stage, the TSWV-YI (CV230505-01) obtained from the Crop Protection Division of the RDA was inoculated into the plants using a modified sap inoculation method based on a previous study [54]. The viruses were first multiplied on *Nicotiana rustica* plants for about one month. Briefly, one gram of the TSWV-infected tobacco leaf was crushed in 10 mL of sodium phosphate buffer (pH 7.0) at a 1:10 *w*/*v* ratio (TSWV-infected sap). The newly developing leaves of the pepper (1–2 main leaves) selected for inoculation were injured with a pin and then sprayed with carborundum. The leaves were rubbed with the TSWV-infected sap and kept in the greenhouse. Five plants per accession were inoculated with the virus, while one plant was maintained as control, which received distilled water instead of TSWV-infected sap. In total, 90 plants were taken for the study, of which 75 were inoculated with the virus, and the other 15 served as a control. RGB and hyperspectral images captured one day before inoculation served as background information.

For four weeks after TSWV inoculation, the pepper leaves were examined weekly for symptom expressions such as mosaic, ring spot, necrosis, and puckered leaf. Subsequently, reverse transcription-PCR was performed on the upper leaves to confirm the presence of the virus in the plants, and finally, to determine whether the pepper accessions were infected with TSWV [54]. Disease incidence analysis followed the following formula.

(1) $$\mathrm{Disease}\;\mathrm{incidence}\;(\%)=\frac{Number\;of\;plants\;infected\;with\;TSWV}{Number\;of\;plants\;innoculated\;with\;TSWV}\times 100$$

### 4.3. Total RNA Extraction and Reverse Transcription-PCR

About 2.0 g of leaf samples were collected from the top leaves of the virus-inoculated plants and stored in 1.5 mL microcentrifuge tubes. The samples were preserved in a negative 80 °C deep freezer until further analysis. The RNA from the leaves was extracted using the Viral Gene-spin^TM^ Viral DNA/RNA Extraction Kit (iNtRON Biotechnology Inc., Sungnam, Republic of Korea) following the manufacturer’s instructions. Subsequently, RT-PCR was carried out using the TSWV-specific primers that amplify 459 bp locus, as described earlier [84]. The forward primer (TSWV-6F): GAGATTCTCAGAATTCCCAGT, and reverse primer (TSWV-6R): AGAGCAATCGTGTCAATTTTATTC were used and the PCR (T100 Thermal Cycler, Bio-Rad, Hercules, CA, USA) reaction comprised 35 cycles, with strand separation at 94 °C for 30 s, an annealing time of 30 s at 60 °C, and an extension time of 1 min at 72 °C. The PCR products were then analyzed on a 1.5% agarose gel and stained with ethidium bromide. After electrophoreses, the gels were visualized under the Gel Doc^TM^, XR+ Imaging System (Bio-Rad, Hercules, CA, USA).

### 4.4. Red–Green–Blue (RGB) and Hyperspectral Image Acquisitions

**Figure 6:**
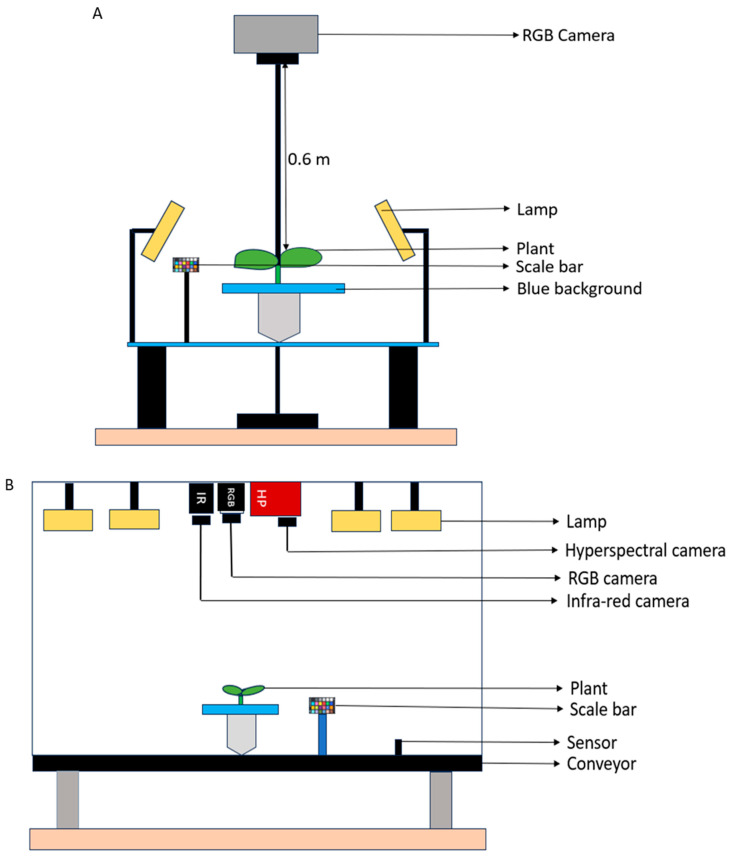
RGB and hyperspectral imaging boxes used for the experiment. (**A**) The RGB imaging set up with a Sony^®^ camera, plants, scale bar, and flashlights. (**B**) Imaging box containing an infra-red camera, RGB camera, hyperspectral camera, and flashlights. A conveyor moves the plant for imaging. A scale bar was included for image segmentation and unit conversion from pixels into mm.

The imaging of the plants was carried out using two imaging systems—RGB using a Sony^®^ camera (Sony Alpha 6000 with Sony E3.5/30 Macro lens, AVCHD 24.3 megapixels, Sony, Tokyo, Japan) and hyperspectral (FX10e, SPECIM, Oulu, Finland). The two images were taken in separate imaging boxes, which were locally assembled (Figure 6). The two imaging boxes were placed in the same room at a distance of about 1.5 m apart.

The imaging room of the RGB was an enclosed area measuring 0.83 m × 0.83 m × 0.83 m. Backlight and light-emitting diode (LED) lights were provided to enhance lighting conditions and to reduce errors that might have been caused by shadows [85]. A Sony^®^ camera was placed about 0.6 m above the blue background and the resolution was adjusted to an area of 210 × 297 mm^2^. The shutter speed was set at 1/10 and the camera focus length was 30.0 mm. A scale was included in the imaging process to help convert values in pixels to millimeters and then later to centimeters. Images were taken at a high precision and then saved on an SD card.

The hyperspectral imaging was taken in a locally built imaging box that housed three camera types, RGB (A7500CG20E, iRAYPLE, Hangzhou, China), infra-red (FLIR A35, FLIR Systems AB, Täby, Sweden), and hyperspectral. The RGB images from the imaging box were only used to assist in analyzing the hyperspectral images. A conveyor moved the plants to the camera positions, where automated photos were captured on a connected computer at a 4 s waiting time.

Images were taken at weekly intervals and 180 images (90 from the RGB set-up and 90 from the imaging box) were taken at every imaging time. In total, 900 RGB and hyperspectral images were acquired and used for the study.

### 4.5. RGB Image Processing

**Figure 7:**
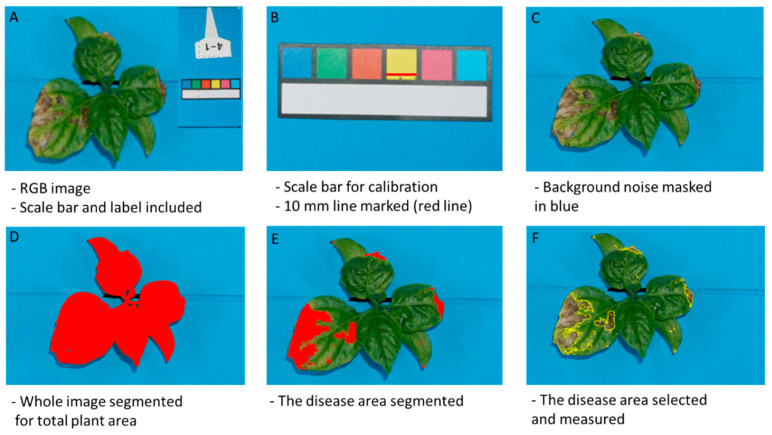
RGB image processing, segmentation, and measurement. (**A**) Raw image from the top view Sony^®^ camera. (**B**) Scale bar to convert units from pixels into mm^2^. (**C**) Image background noise removed. (**D**) Total leaf area masked in red. (**E**) Disease leaf area segmented as a red mask. (**F**) Disease area selected for measurement.

The RGB images were analyzed using the ImageJ software version 1.52a. After uploading the image onto the ImageJ platform, a scale was set to convert measurements from pixels into millimeters using the scale bar (Figure 7).

Background noise was separated following the steps: edit→ selection → make inverse → edit → fill. Then the total leaf area was segmented using the YUV color separation model set at Y, 0, 255, U, 0, 132 on average, and V, 0, 255. The ROI manager measured the total leaf area in mm^2^ which was later converted into cm^2^. We further measured the infected area setting Y at 0 low, 255 high, U at 0 low and an average of 127–132 high, and V at 127–135 low and 255 high, depending on the sample. These steps were followed for the individual plants. In total, 450 individual RGB images were analyzed.

Data acquisition followed up with disease severity analysis following the formula:

(2) $$\mathrm{Disease}\;\mathrm{severity}\;(\%)=\frac{Disease\;area\;from\;top\;view\;RGB\;image\;segmentation}{Total\;plant\;area\;from\;top\;view\;RGB\;image\;segmentation}\times 100$$

### 4.6. Hyperspectral Image Processing

**Figure 8:**
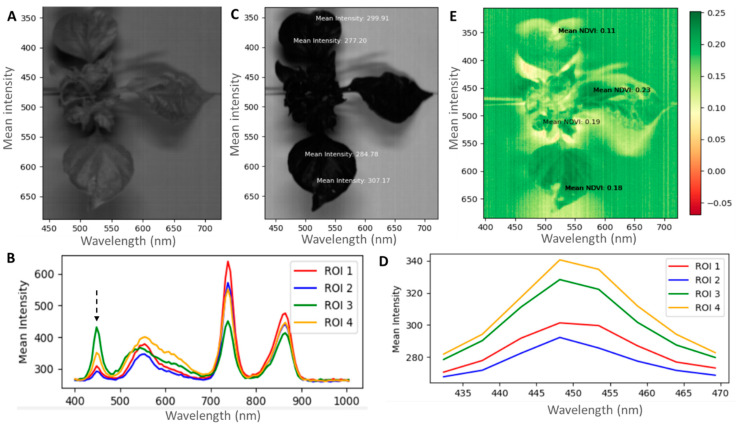
Hyperspectral image analysis process. (**A**,**B**)—band detection, (**C**,**D**)—intensity of TSWV infected parts of the plant, and (**E**)—NDVI plot showing areas affected by the virus based on a scale (green indicating low intensity and red indicating high intensity). The black arrow shows the band spot where the TSWV infection was assumed to be detected.

The hyperspectral file was analyzed using a lab-developed Python-based hyperspectral image analysis software (using Python version 3.12—not yet publicly released). First, the images were subjected to a wavelength between 400.93 and 1004.5 nm to identify the band range for TSWV detection (Figure 8). After a 430–470 nm detection, the wavelength dimensions were limited to the range, and about four to five spots were selected for the reflectance intensity.

Also, the normalized difference vegetative index (NDVI) model was used to measure the severity of damage based on the different symptoms of the TSWV. NDVI was computed as the differences between the selected wavelengths (near-infrared (NIR)) and red (RED) reflectance, divided by the sum of the wavelengths [59].

(3) $$\mathrm{NDVI}=\frac{W2-W1}{W2+WI}$$

Like the reflectance intensity, the NDVI images were acquired using the software and then 4 to 5 disease spots on the leaves were picked for NDVI determination, where W2 is the near-infrared (NIR) and W1 is the red (R) band reflectance.

### 4.7. Statistical Analysis

After data segmentation, the results from the imaging were analyzed using the linear mixed effect model in the R statistical package version 4.4.0. In the model, accessions and time after infection (weeks) were used as fixed factors, while the number of plants for data collection was treated as a random factor. Also, analyses were carried out comparing the different species and the three levels of severity of TSWV. These species and severity levels were used in separate models as fixed factors with time, while the number of plants used for data collection was used as a random factor. The normality test was conducted with quantile-quantile plots and normal distribution tables. This way, total leaf area, disease leaf area, and percentage severity were log-transformed. Significant differences were checked with the analysis of variance (ANOVA) function. Post hoc analysis was applied with Tukey’s honestly significant difference test. The data was visually presented using the ggplot2 package in the R software.

## 5. Conclusions

TSWV is a serious viral disease in pepper production. We explored RGB and hyperspectral imaging approaches to study the inter- and intra-severity of damages of TSWV to pepper germplasms and the technique for identifying different symptoms of TSWV in pepper accessions. In bioassay for TSWV infection, some accessions, including *C. chinense*, showed a low disease incidence (0–40%). Growth in terms of canopy spread and expansion of disease areas were studied using RGB and hyperspectral imaging. Different growth responses and disease symptoms were identified in the pepper germplasms studied. As shown by RGB imaging, the severity of TSWV ranged from low (0–0.5%), to moderate (0.5–1.0%), and to high (above 1.0%) among the germplasms. The *C. baccatum* species had the highest severity of TSWV, while the *Capsicum* sp. and the *C. chinense* species demonstrated the lowest severity. Common symptoms identified among all the accessions included mosaic, necrosis, ring spots, puckered leaves, and stuntedness. All the symptoms can be studied with precision using the imaging techniques. However, puckered leaves and stunted growth may need special attention in the imaging process. The hyperspectral validation process proved effective and precise in quantifying the severity of infection. The visible light wavelength of 430–470 nm detected the differences between the infected and normal parts of the leaves of different pepper accessions. The study of the leaves within the 430 to 470 nm wavelength showed intensities as high as 430 for the infected parts, but as low as 270 for the healthy parts. On the other hand, NDVI values for the normal leaves were high, ranging from 0.21 to 0.23, but were around 0.14–0.19 for the infected parts. The RGB and the hyperspectral imaging techniques can be used for the accurate and precise measurement of the severity of the disease during different stages of plant growth and development. We suggest combining the three assessment techniques for plant disease studies—RGB for visual observation of infected parts or symptoms, hyperspectral for spectral signatures of the pathogenic activity, and bioassay to confirm the presence of the pathogen in the plant tissues.

We recommend the expansion of the imaging techniques to field studies for host–vector interactions and other abiotic factors (such as climatic conditions), to screen out plants’ resistance to the disease in different environmental conditions, and the application of a farmer-friendly app on smartphones for easy monitoring of the disease at the farm level.

## Figures and Tables

**Table 1:** Bioassay results of pepper germplasms after infection with the TSWV-YI.

Accession Number	Scientific Name	Accession Name	Origin ^a^	Status	Common Symptoms ^b^	RT-PCR ^c^	Disease Incidence ^d^ (%)
IT308738	*C. chinense*	Chi 16/1026-1	HUN	Breeding line	-	−	0
IT308753	*C. chinense*	Chi 39/1055	HUN	Breeding line	-	−	0
IT284050	*C. chinense*	Grif 9273	CRI	- ^e^	m, pl	++	40
IT136625	*Capsicum* sp.	Pathari Local	NPL	Landrace	m	++	40
IT284034	*C. baccatum*	PI 633753	PRY	Landrace	n, pl	++++	80
IT284059	*C. chinense*	PI 260524	PER	-	n	++++	80
IT284069	*C. chinense*	PI 653674	COL	-	m, pl	++++	80
IT270672	*C. baccatum*	AC12-175	BRA	Landrace	n, pl	++++	80
IT136615	*Capsicum* sp.	Chatar Local	NPL	Landrace	n, pl	++++	80
IT158568	*C. frutescens*	C02424	-	-	n	++++	80
IT136626	*Capsicum* sp.	Pakhribas 2 Local	NPL	Landrace	n	++++	80
IT218962	*C. frutescens*	TC05779	MEX	-	n	++++	80
IT32436	*C. annuum*	Jindo Jaerae	KOR	Landrace	n, pl	+++++	100
IT164927	*C. frutescens*	Legon 18	-	-	n	+++++	100
IT103079	*C. annuum*	Dwaeji gochu	KOR	Landrace	rs, pl	+++++	100

^a^ Country codes (ISO 3166-1, alpha-3). ^b^ m, mosaic; pl, puckered leaf; n, necrosis; rs, ring spot; -, no symptom. ^c^ ‘−’, negative to TSWV-YI; ‘+’, number of plants positive to TSWV-YI. ^d^ (Number of plants infected with TSWV)/(Number of plants inoculated with TSWV) × 100. ^e^ No data.

**Table 2:** Disease severity percentages and status of the accessions based on RGB imaging results.

Accession Number	Scientific Name	Accession Name	Disease Severity ^a^ (%)	SE ^b^	Df ^c^	Lower. CL ^d^	Upper. CL	Disease Severity Status ^e^
IT308738	*C. chinense*	Chi 16/1026-1	0.00	0.00	4	0.00	0.00	Low
IT308753	*C. chinense*	Chi 39/1055	0.00	0.00	4	0.00	0.00	Low
IT218962	*C. frutescens*	TC05779	0.14	0.29	4	−0.66	0.94	Low
IT284034	*C. baccatum*	PI 633753	0.56	0.39	4	−0.54	1.66	Moderate
IT284050	*C. chinense*	Grif 9273	0.67	0.42	4	−0.50	1.85	Moderate
IT136626	*Capsicum* sp.	Pakhribas-2 Local	0.72	0.44	4	−0.49	1.93	Moderate
IT136615	*Capsicum* sp.	Chatar Local	0.75	0.44	4	−0.48	1.98	Moderate
IT136625	*Capsicum* sp.	Pathari Local	0.75	0.44	4	−0.48	1.99	Moderate
IT103079	*C. annuum*	Dwaeji gochu	1.03	0.51	4	−0.40	2.46	High
IT284069	*C. chinense*	PI 653674	1.32	0.54	4	−0.17	2.82	High
IT32436	*C. annuum*	Jindo Jaerae	1.35	0.59	4	−0.30	3.00	High
IT164927	*C. frutescens*	Legon 18	1.45	0.62	4	−0.27	3.17	High
IT270672	*C. baccatum*	AC12-175	1.82	0.71	4	−0.16	3.80	High
IT284059	*C. chinense*	PI 260524	1.82	0.71	4	−0.16	3.80	High
IT158568	*C. frutescens*	C02424	5.68	1.69	4	0.99	10.38	High

^a^ (Disease area from top-view RGB image segmentation)/(Total plant area from top-view RGB image segmentation) × 100; ^b^ SE = standard error; ^c^ Df = degree of freedom; ^d^ CL = confidence level; ^e^ accessions with disease severity percentages from 0.0 to 0.5% were low, between 0.5 and 1.0% were moderate, and above 1.0% were high.

## Data Availability

Data will be available upon request.

## Supplementary Materials

The following supporting information can be downloaded at: https://www.mdpi.com/article/10.3390/plants13233447/s1. The original images used in Figure 5 for RGB and hyperspectral imaging of TSWV-YI common symptoms in pepper germplasms are provided.

## Appendix A

**Table A1:** Probability level of the accessions, species, plant appearance status and time to canopy spread, disease area, and percentage severity from RGB images analysis of top-view camera.

Variations	Canopy Spread	Disease Area	Percentage Severity
Accessions	0.0001	0.0001	0.0001
Time	0.0001	0.0001	0.0001
Accessions × Time	0.0001	0.0001	0.0001
Species	0.0001	0.0001	0.0001
Time	0.0001	0.0001	0.0009
Species × Time	0.0001	0.0001	0.0472
Severity Status	0.0024	0.0001	0.0001
Time	0.0001	0.0001	0.0001
Severity Status × Time	0.0072	0.0001	0.0001
