# Supplementary figures and images for: Exploring Imaging Techniques for Detecting Tomato Spotted Wilt Virus (TSWV) Infection in Pepper (Capsicum spp.) Germplasms
Source: Plants (Basel). 2024 Dec 9;13(23):3447. doi: 10.3390/plants13233447 (PMC11644830; doi:10.3390/plants13233447)

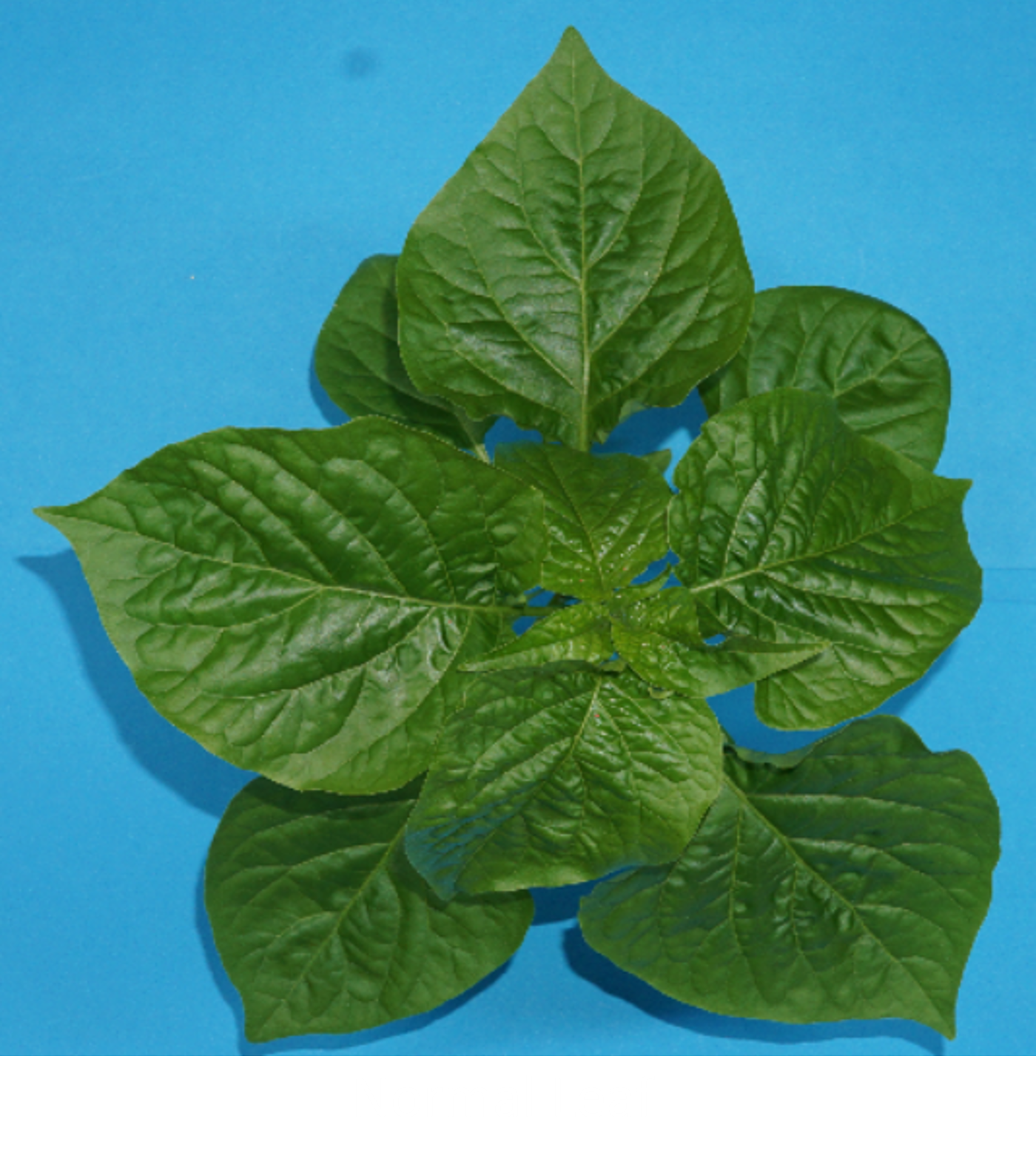

Supplement: Supplementary file 1 [file plants-13-03447-s001.zip › 1. Normal leaf.png]

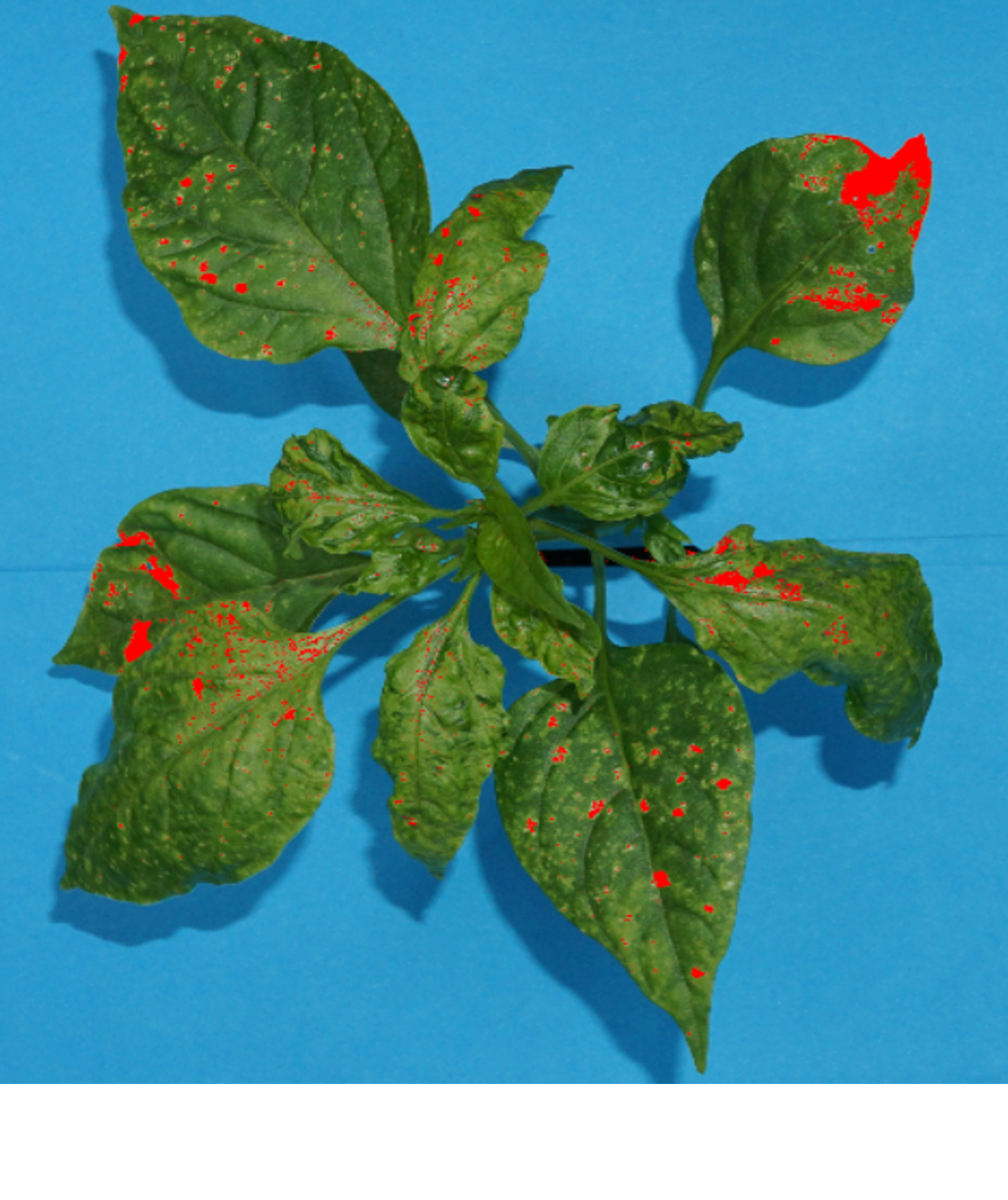

Supplement: Supplementary file 1 [file plants-13-03447-s001.zip › 10. Rings pot RGB.png]

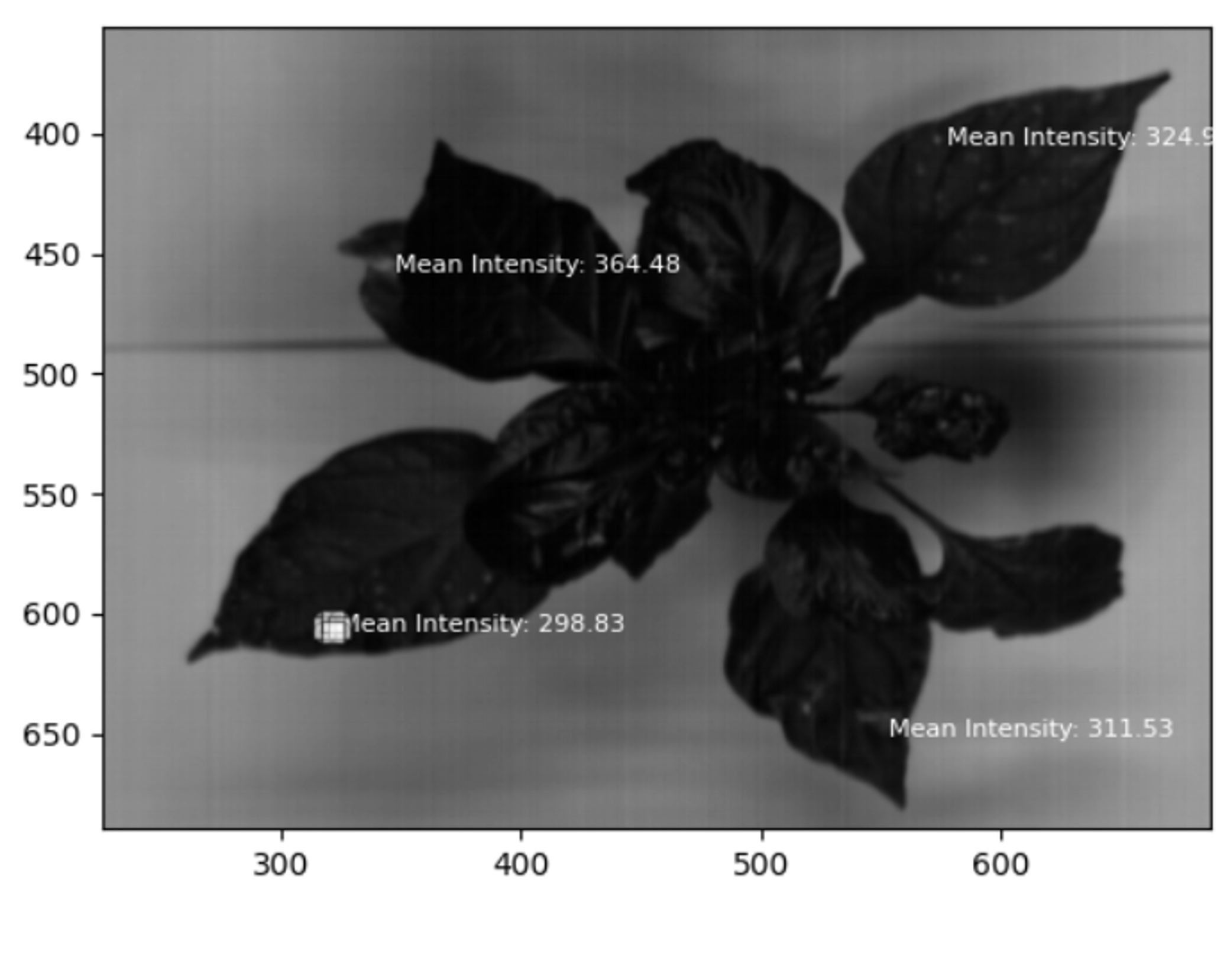

Supplement: Supplementary file 1 [file plants-13-03447-s001.zip › 11. Ring spot hyperspectral.png]

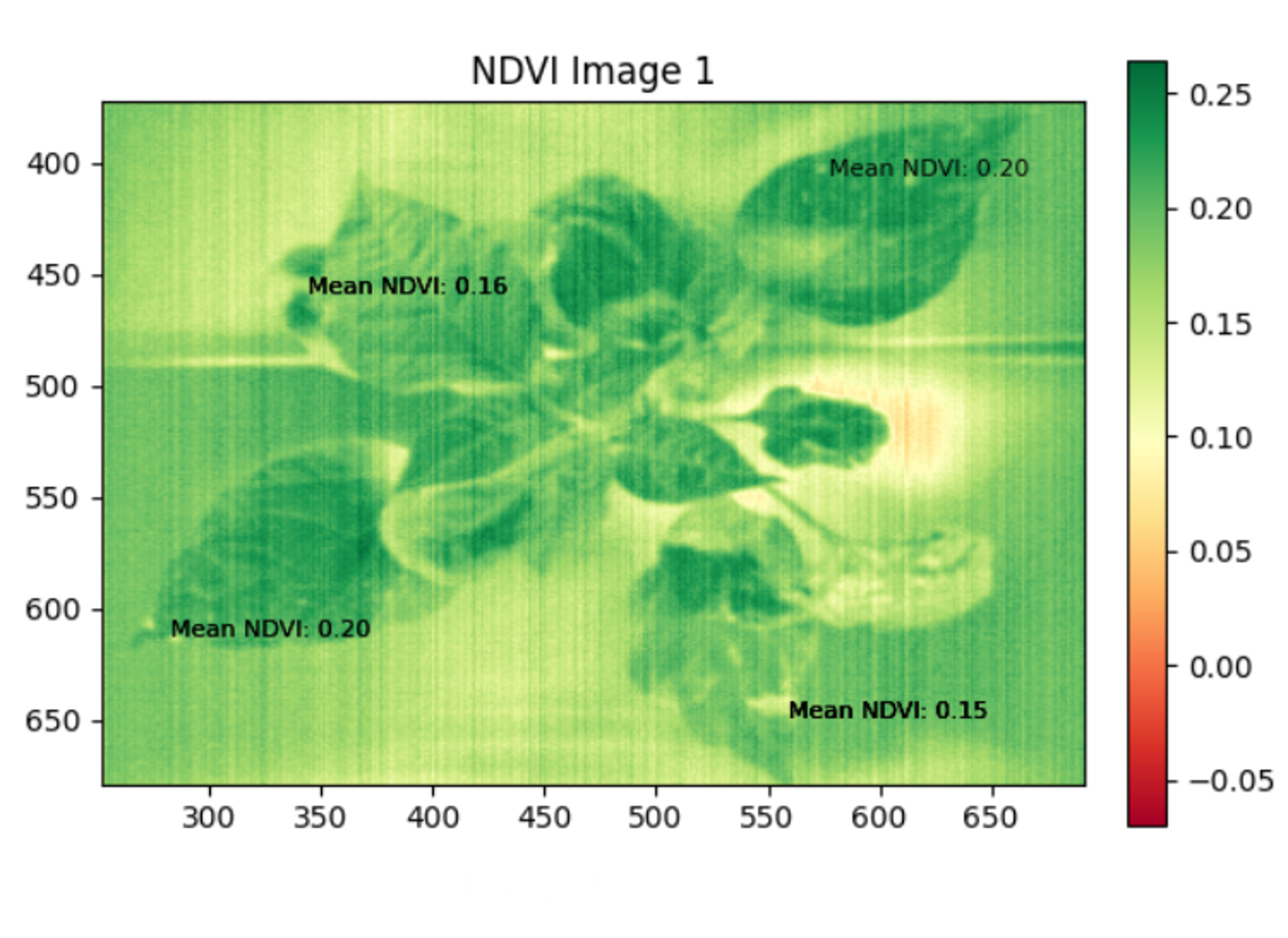

Supplement: Supplementary file 1 [file plants-13-03447-s001.zip › 12. Ring spot NDVI.png]

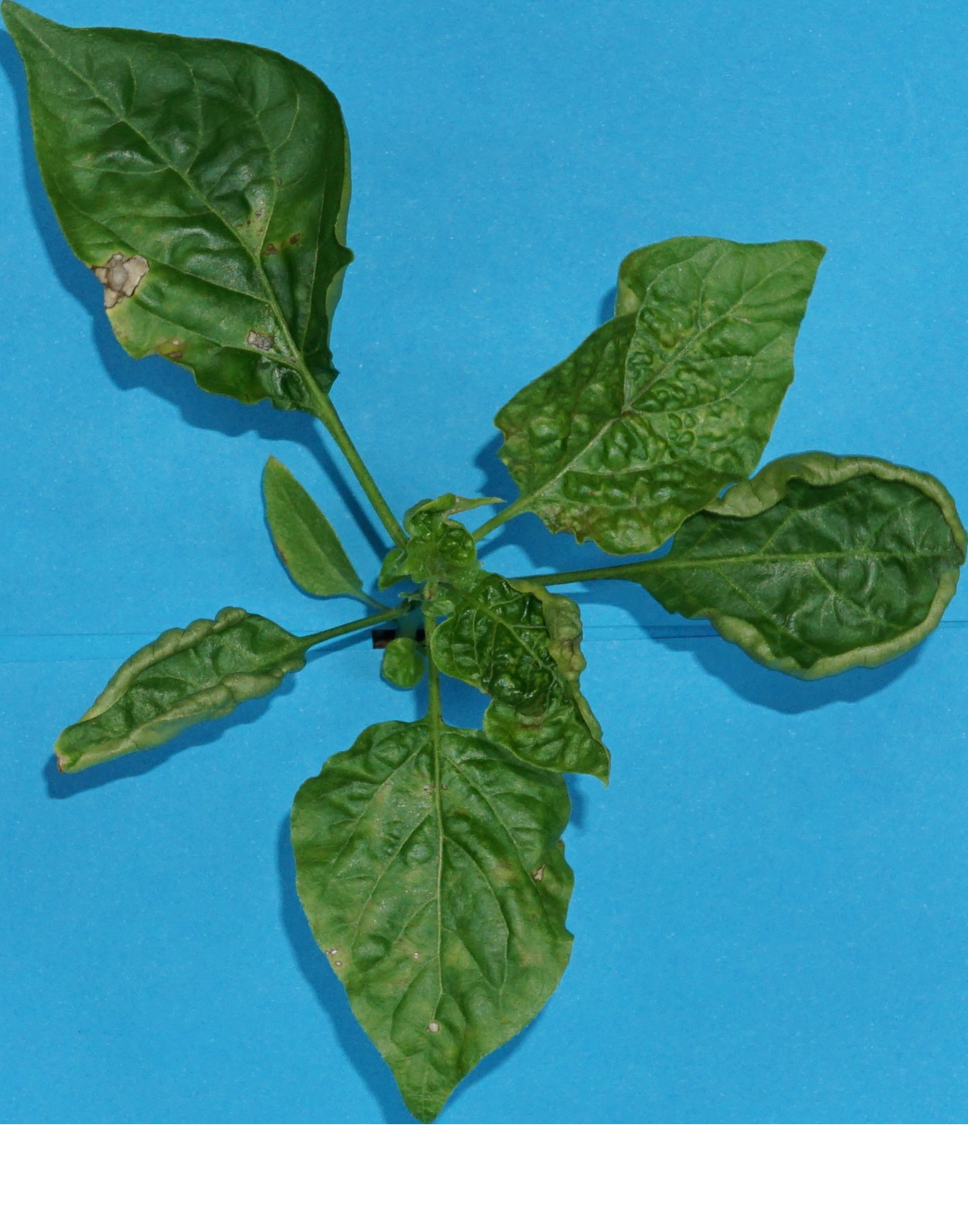

Supplement: Supplementary file 1 [file plants-13-03447-s001.zip › 13. Puckered leaves.png]

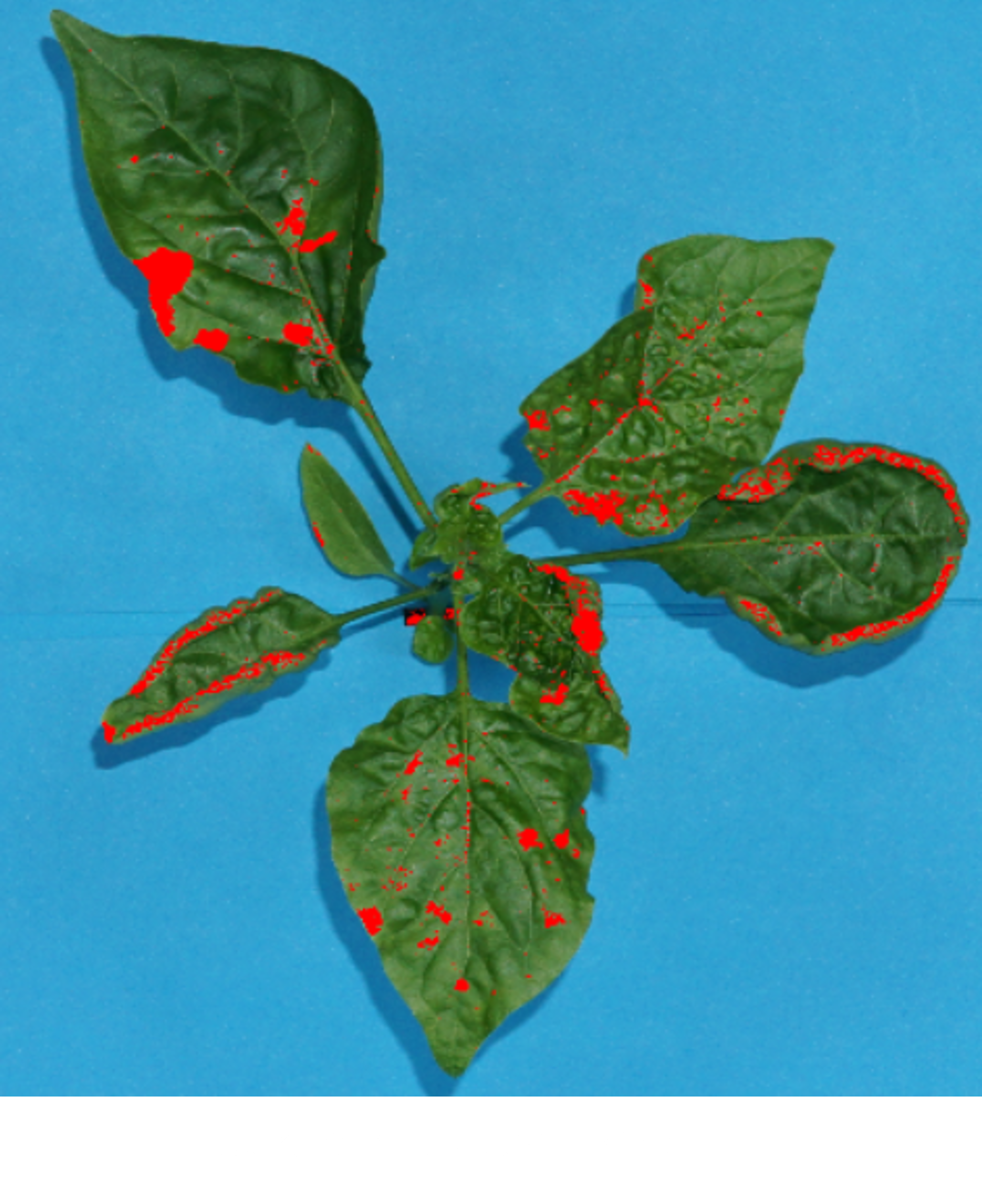

Supplement: Supplementary file 1 [file plants-13-03447-s001.zip › 14. Puckered leaves RGB.png]

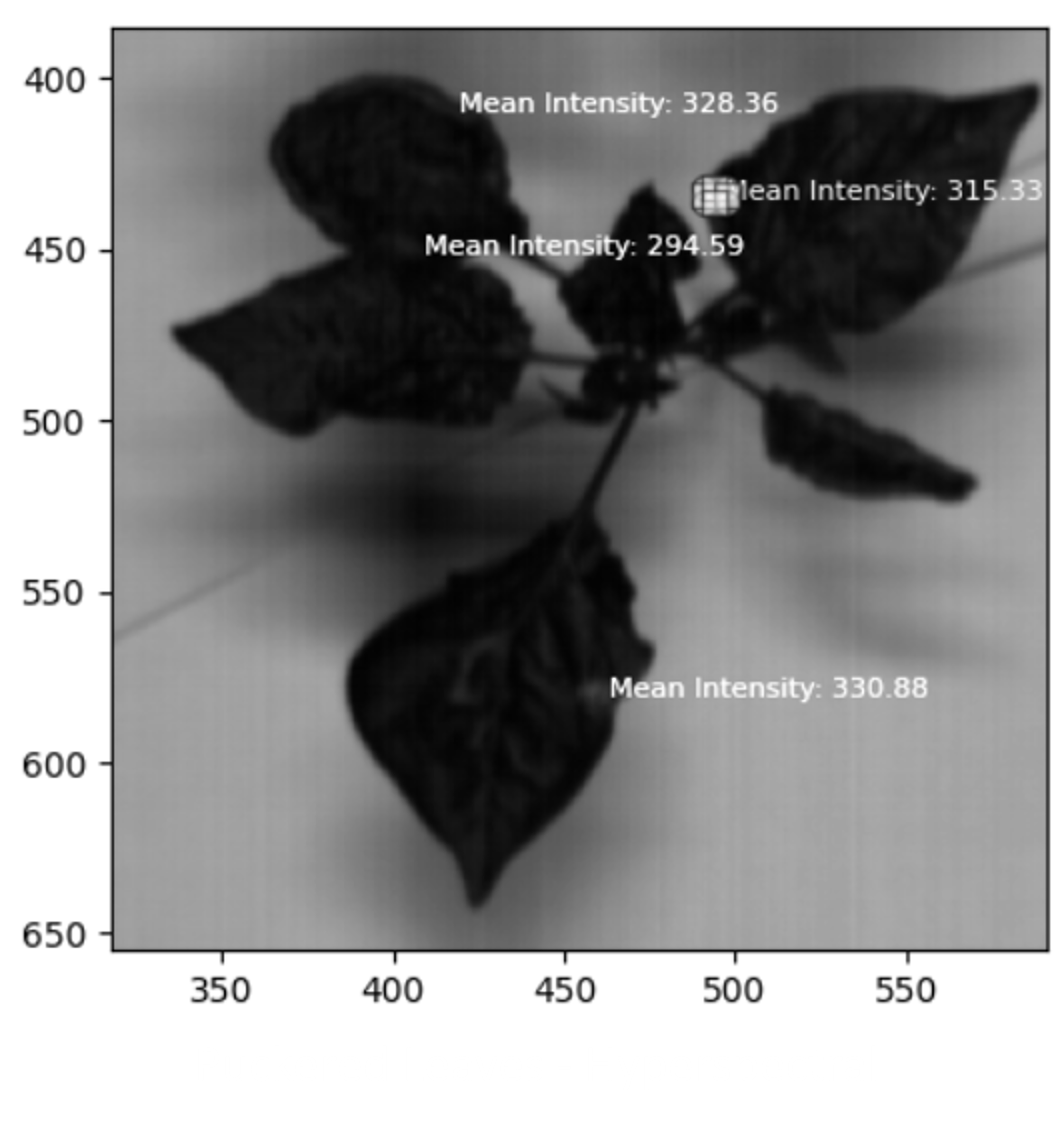

Supplement: Supplementary file 1 [file plants-13-03447-s001.zip › 15. Puckered leaves hyperspectal.png]

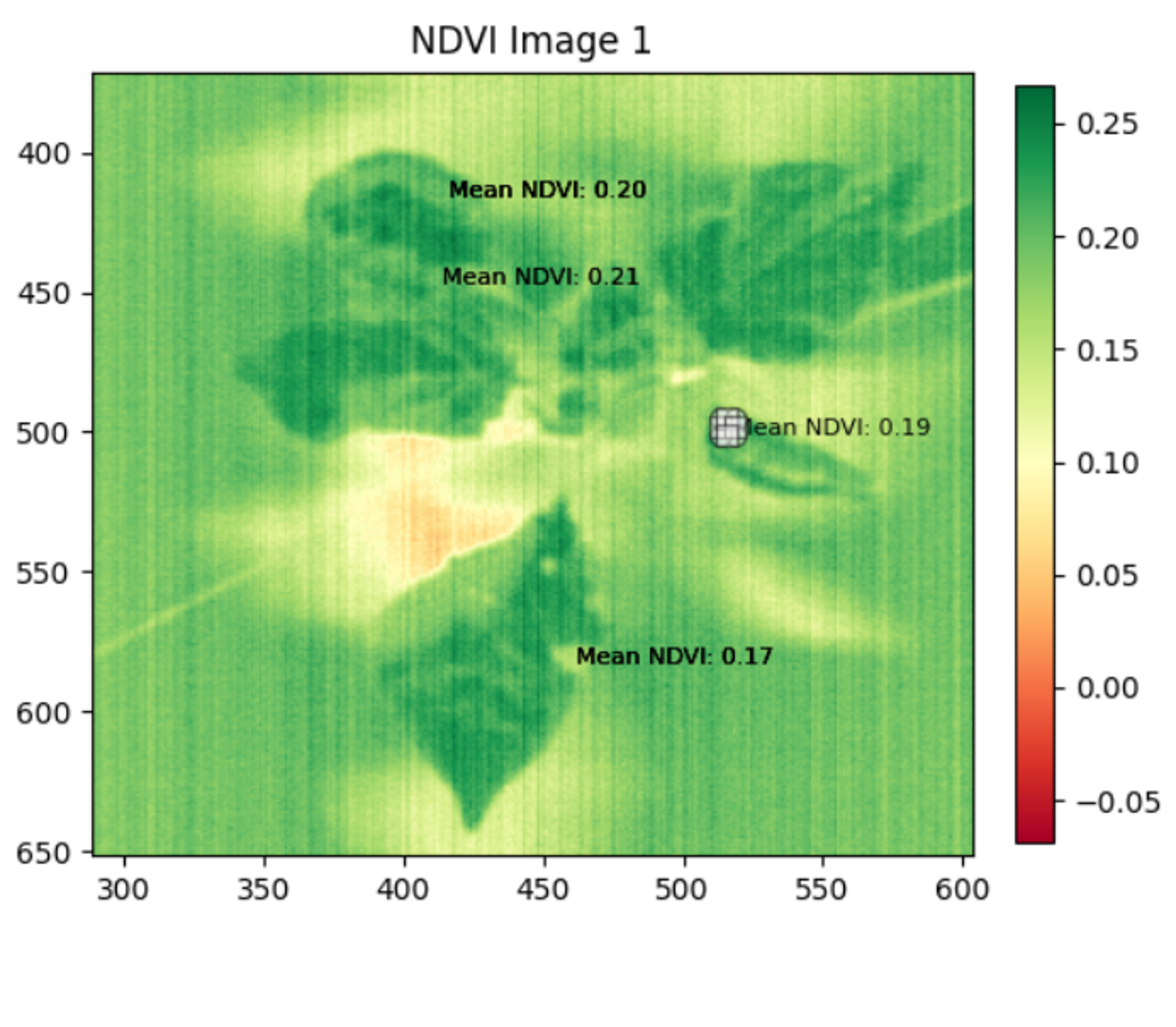

Supplement: Supplementary file 1 [file plants-13-03447-s001.zip › 16. Puckered leaves NDVI.png]

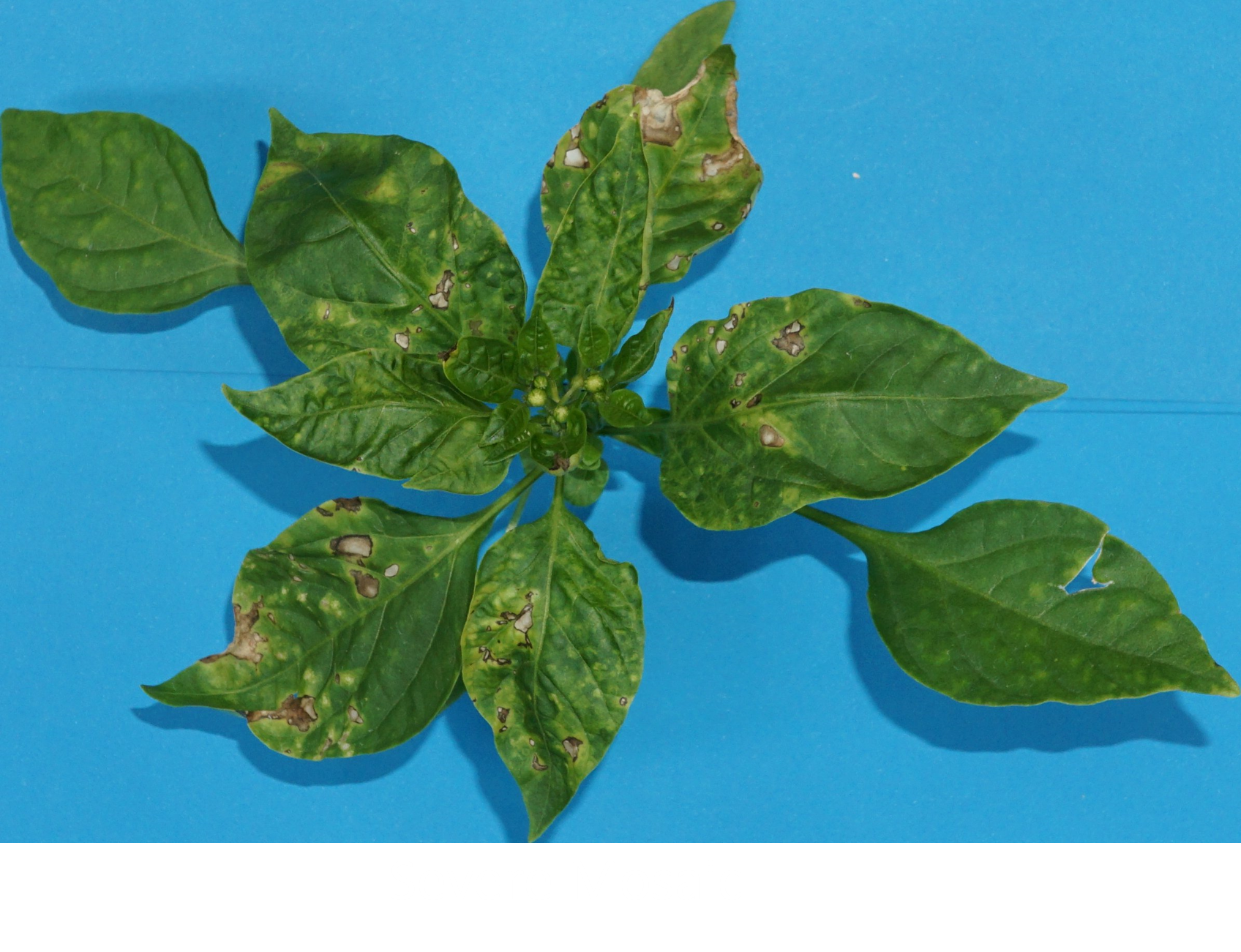

Supplement: Supplementary file 1 [file plants-13-03447-s001.zip › 17. Severe mosaic.png]

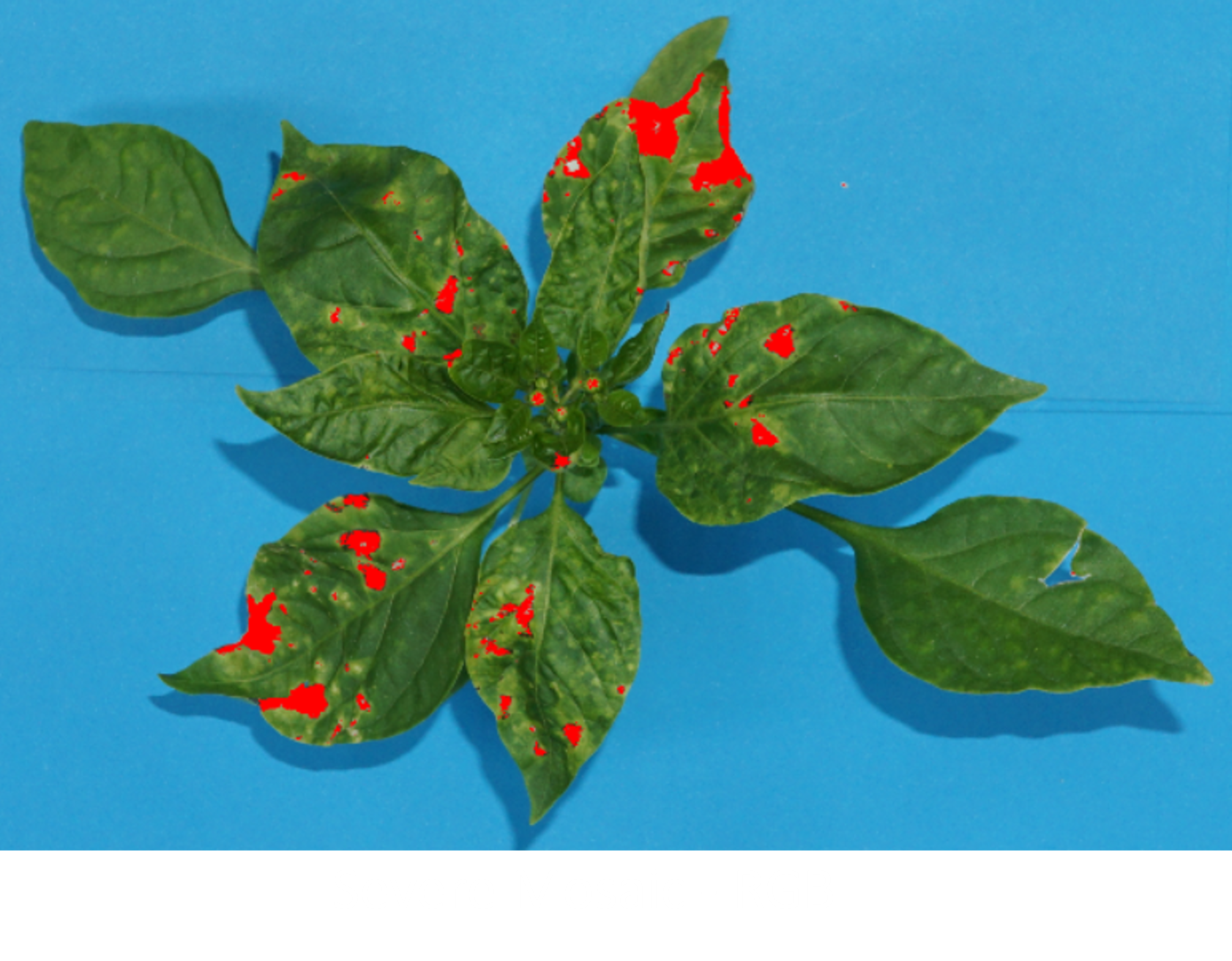

Supplement: Supplementary file 1 [file plants-13-03447-s001.zip › 18. Severe mosaic RGB.png]

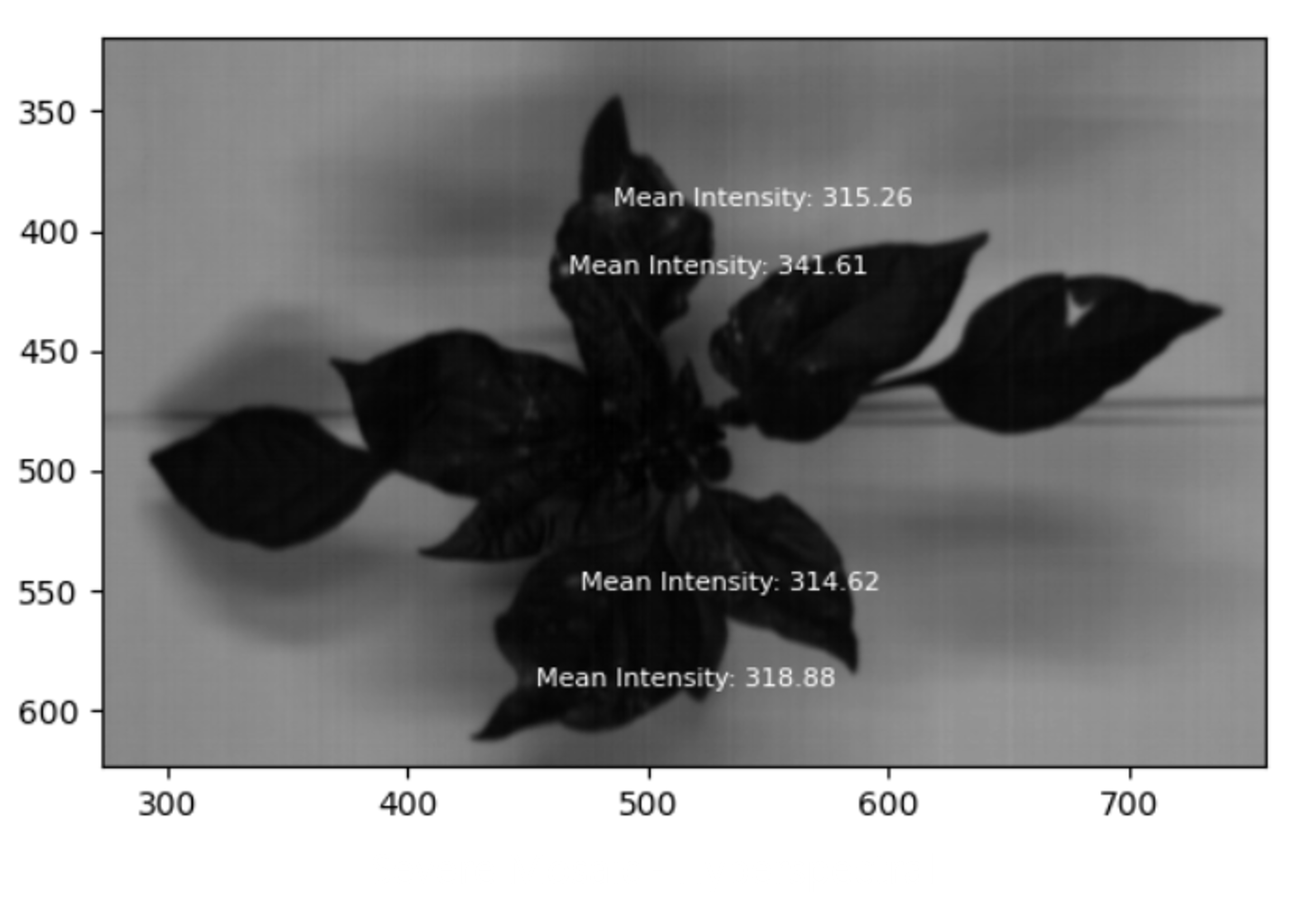

Supplement: Supplementary file 1 [file plants-13-03447-s001.zip › 19. Severe mosaic hyperspectral.png]

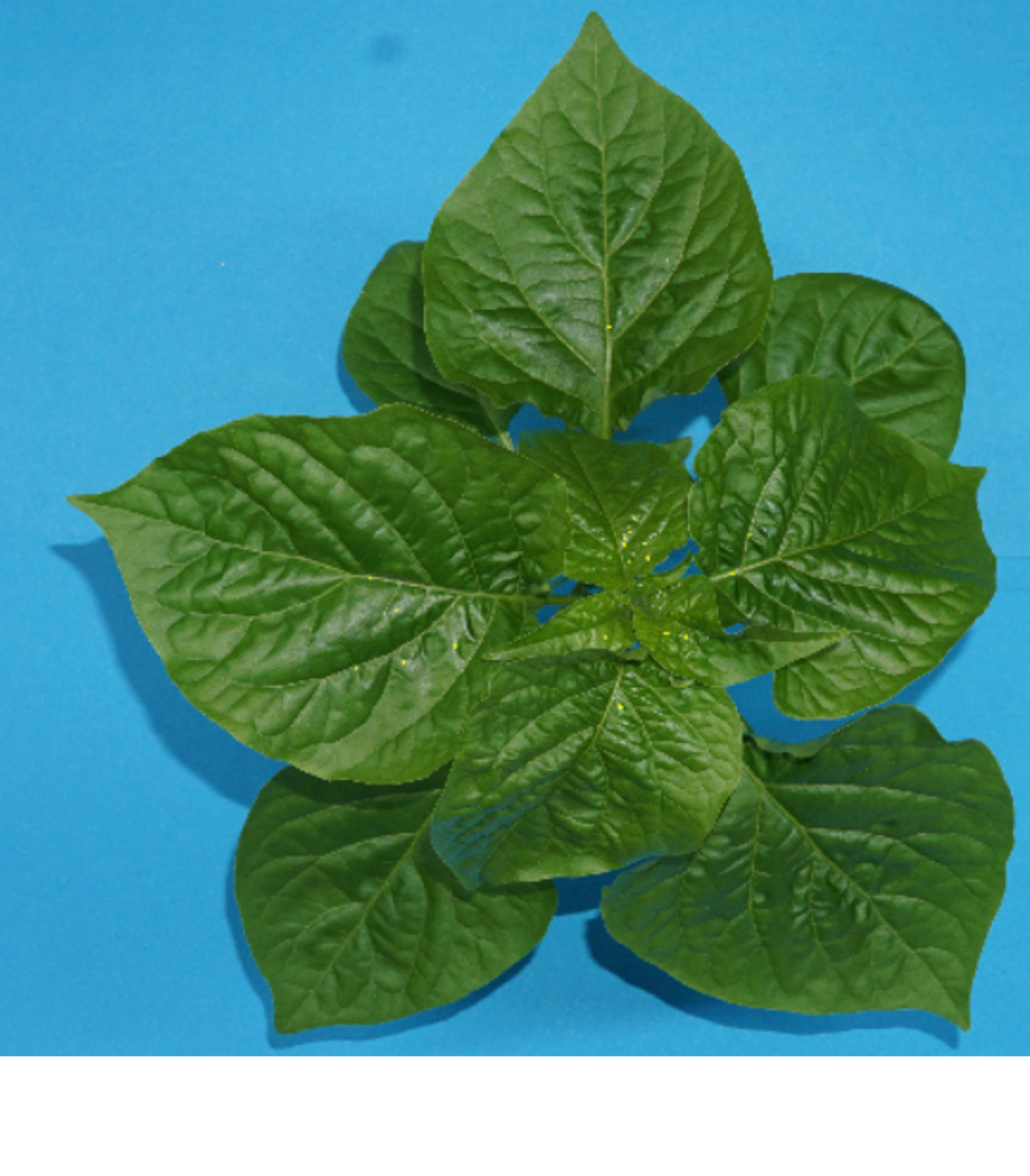

Supplement: Supplementary file 1 [file plants-13-03447-s001.zip › 2. Normal leaf RGB.png]

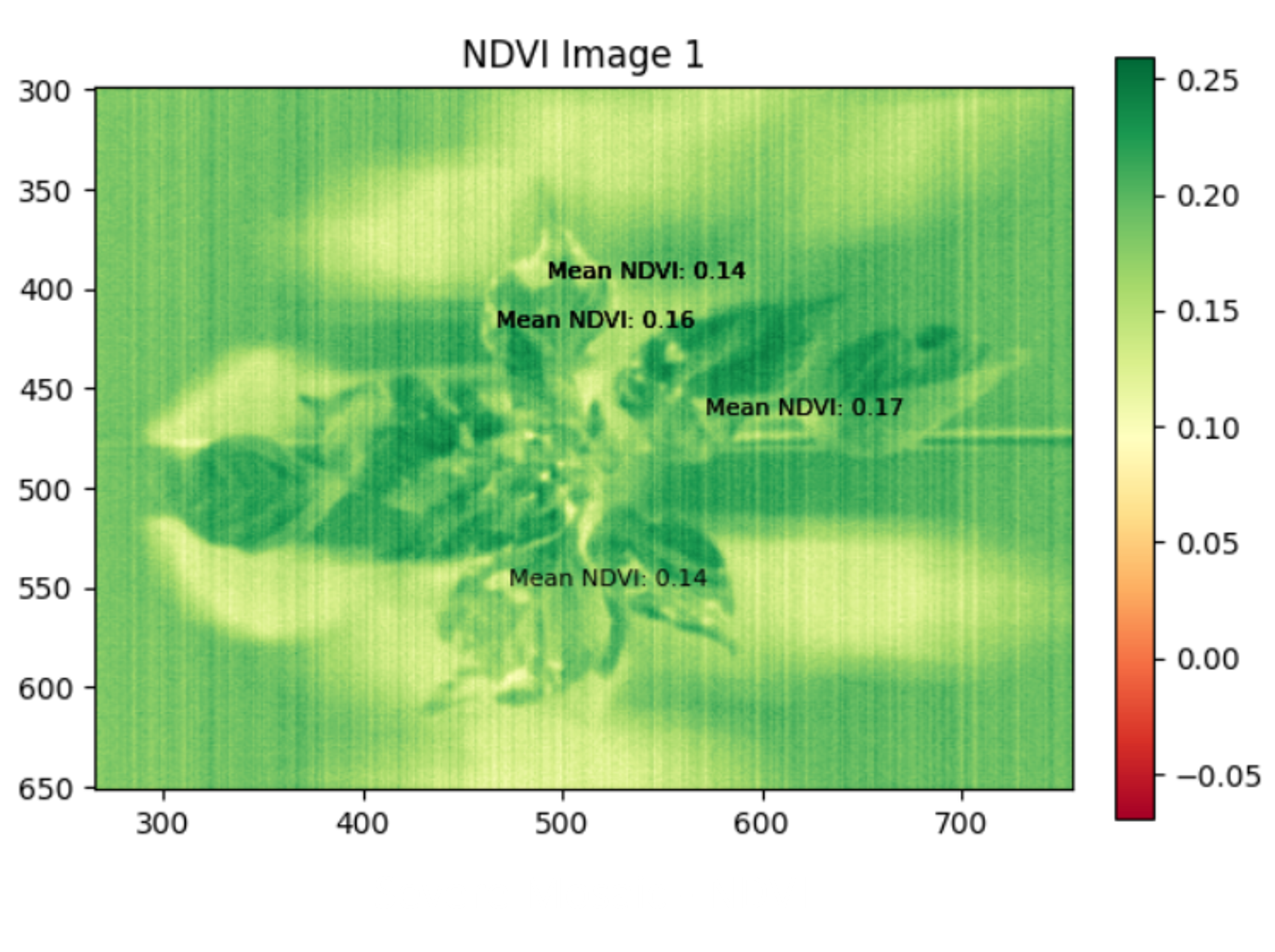

Supplement: Supplementary file 1 [file plants-13-03447-s001.zip › 20. Severe mosaic NDVI.png]

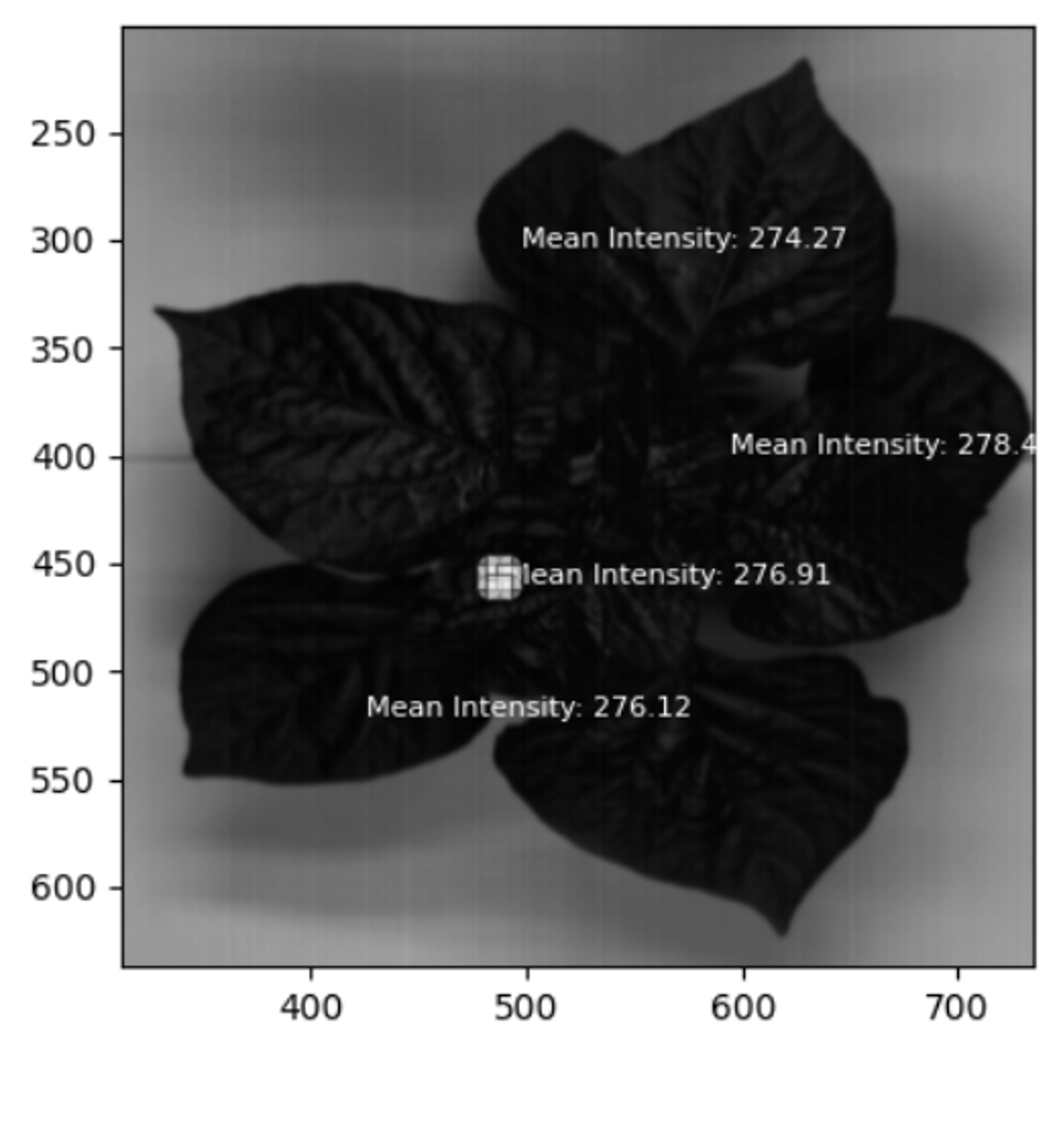

Supplement: Supplementary file 1 [file plants-13-03447-s001.zip › 3. Nornal leaf hypersepctral.png]

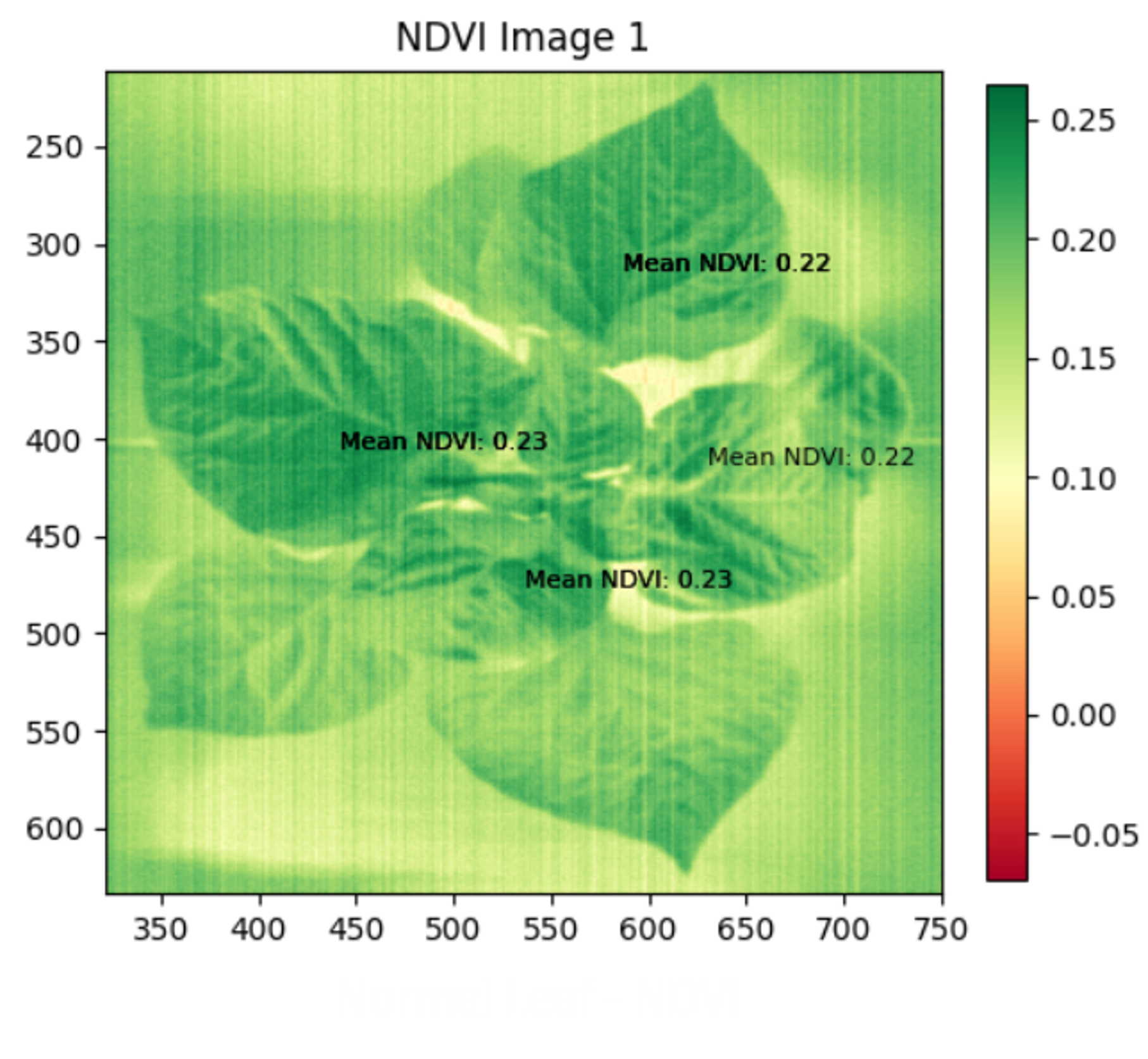

Supplement: Supplementary file 1 [file plants-13-03447-s001.zip › 4. Normal leaf NDVI.png]

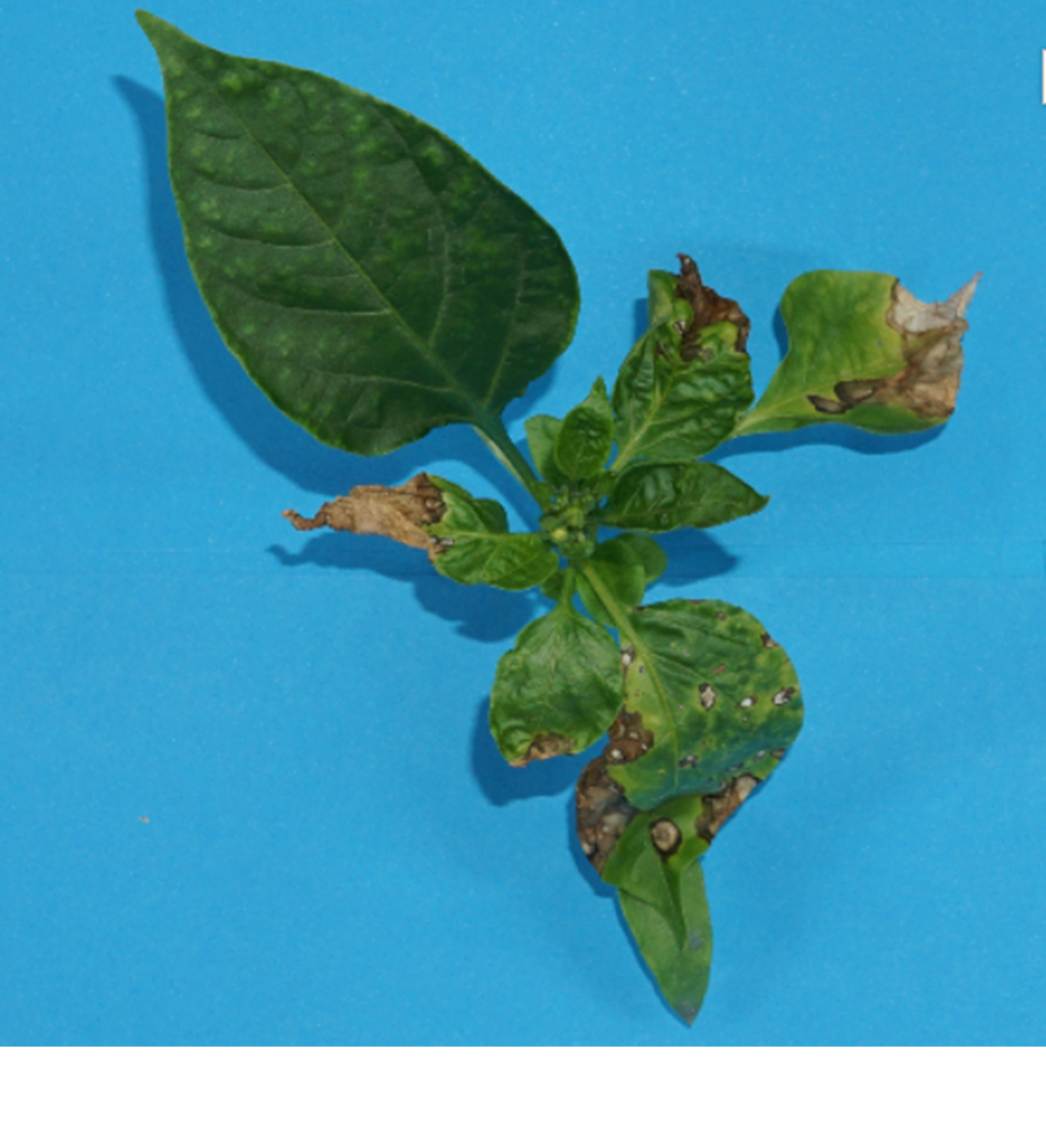

Supplement: Supplementary file 1 [file plants-13-03447-s001.zip › 5. Necrosis.png]

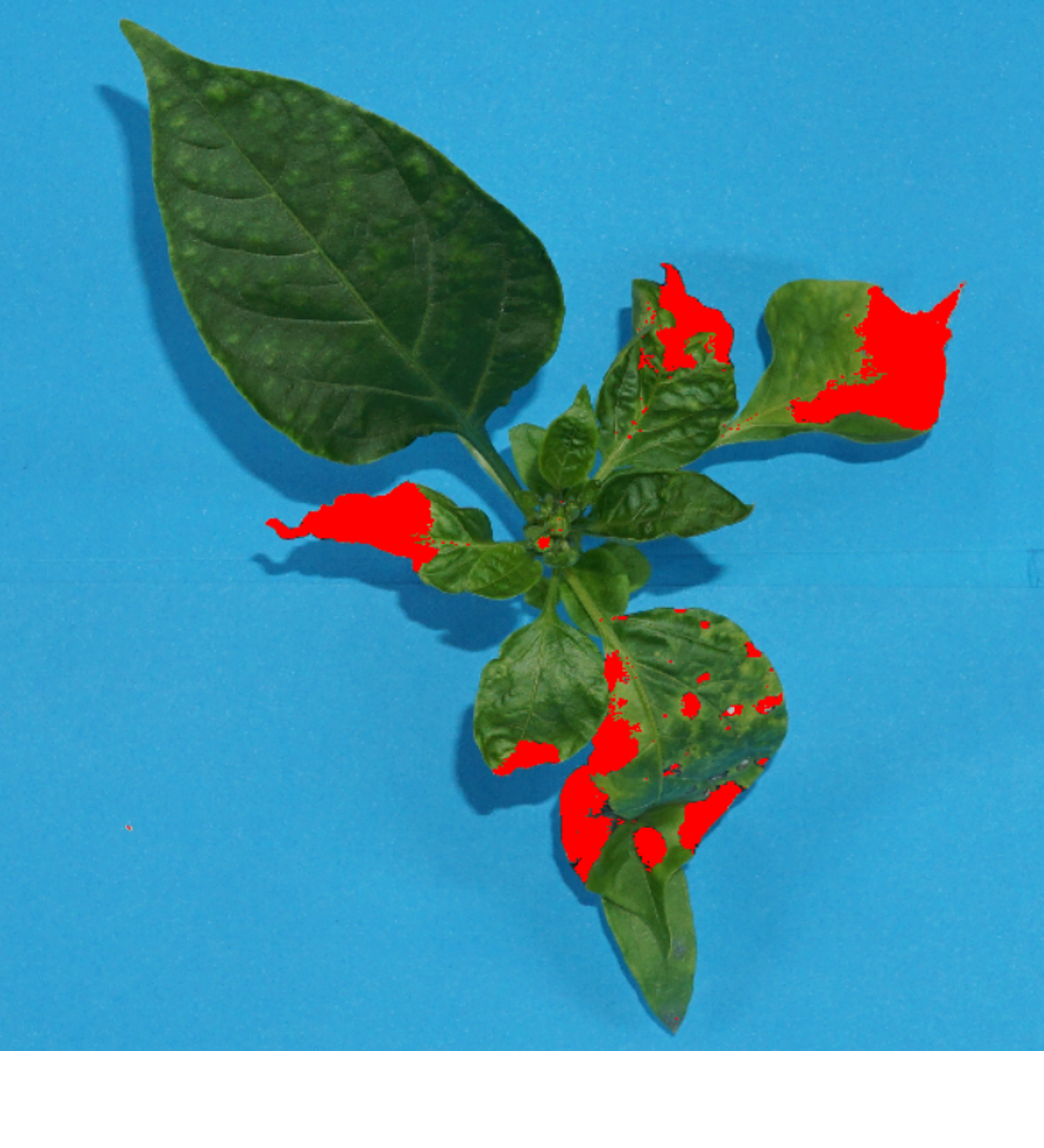

Supplement: Supplementary file 1 [file plants-13-03447-s001.zip › 6. Necrosis - RGB.png]

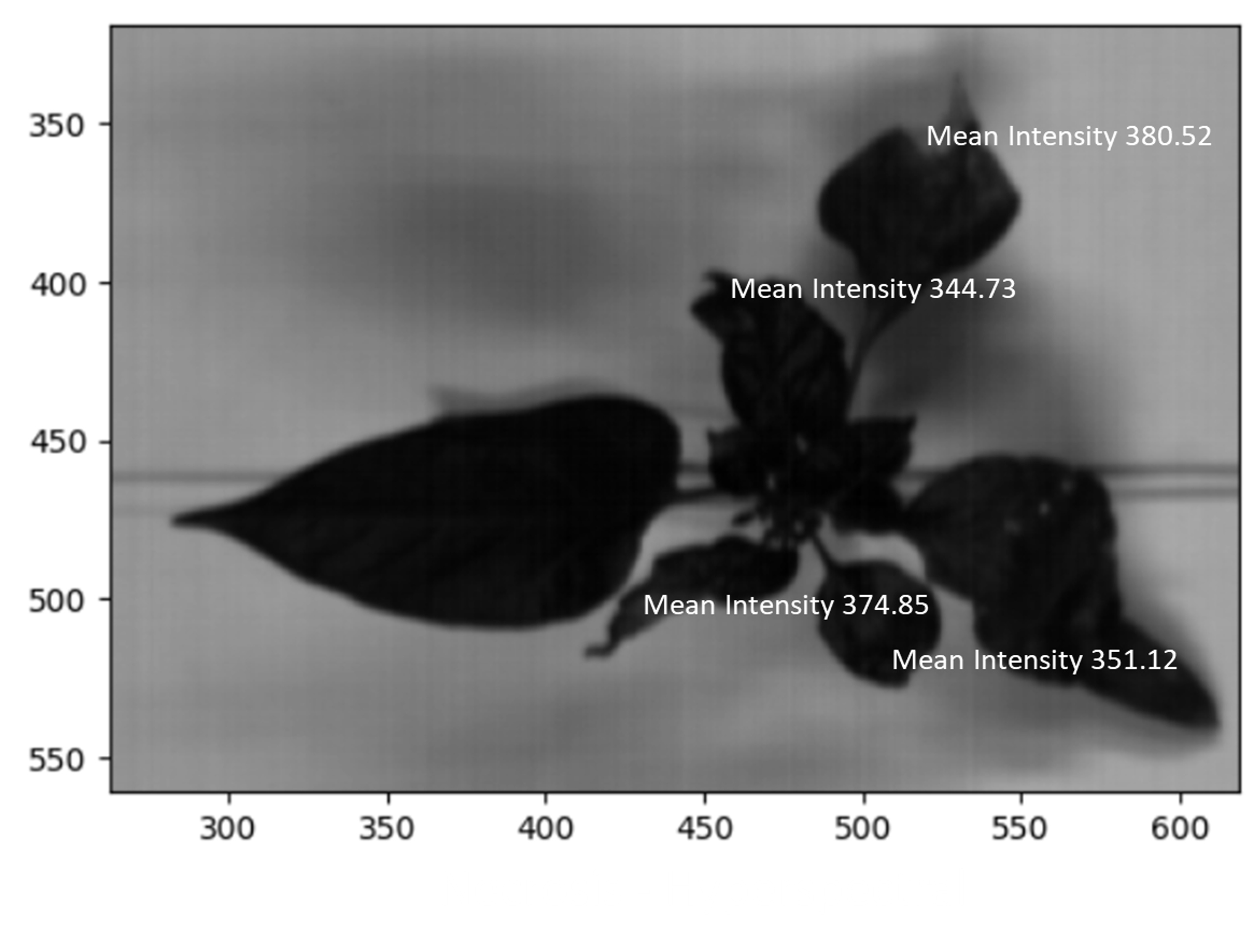

Supplement: Supplementary file 1 [file plants-13-03447-s001.zip › 7. Necrosis - hyperspectral.png]

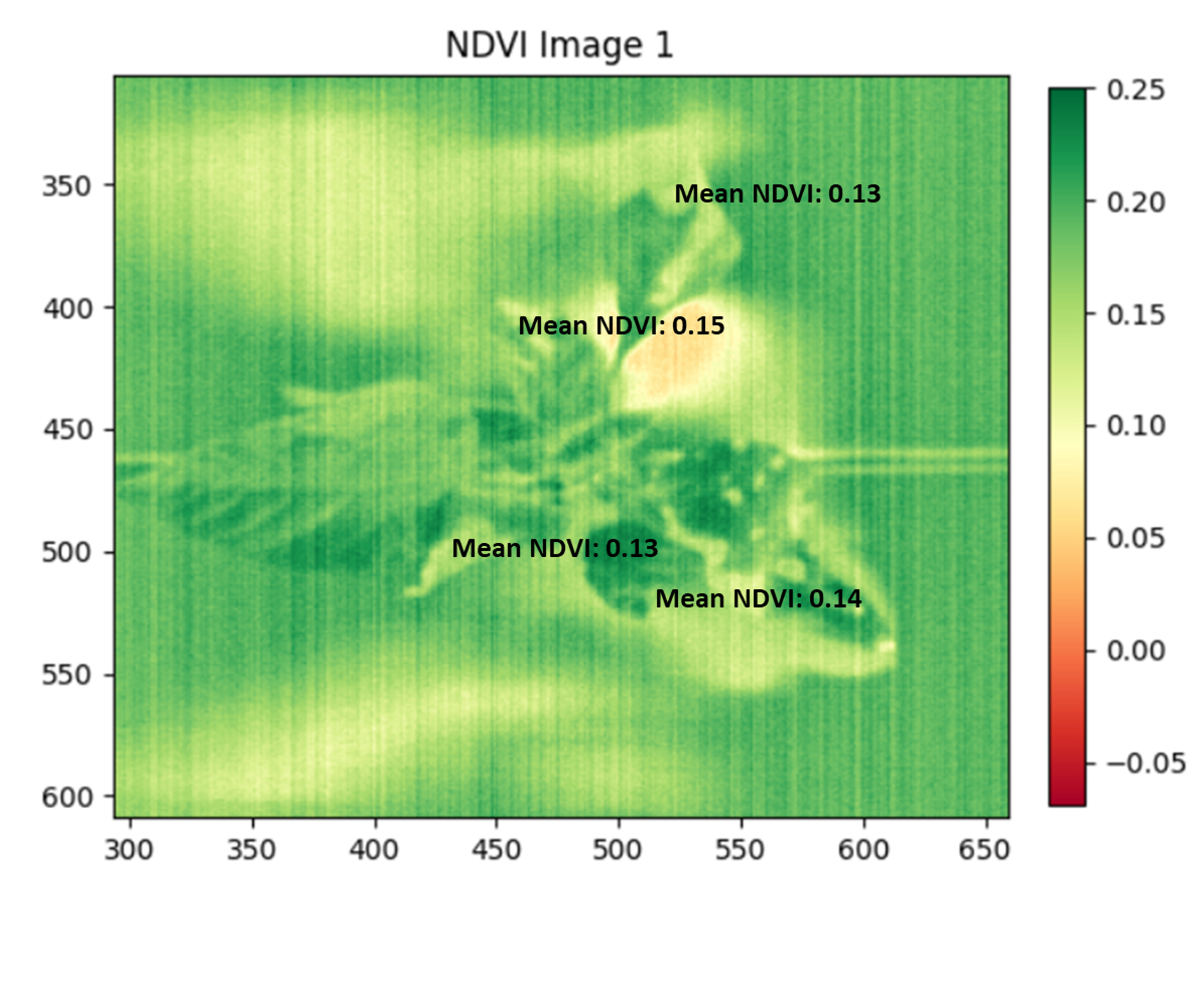

Supplement: Supplementary file 1 [file plants-13-03447-s001.zip › 8. Necrosis NDVI.png]

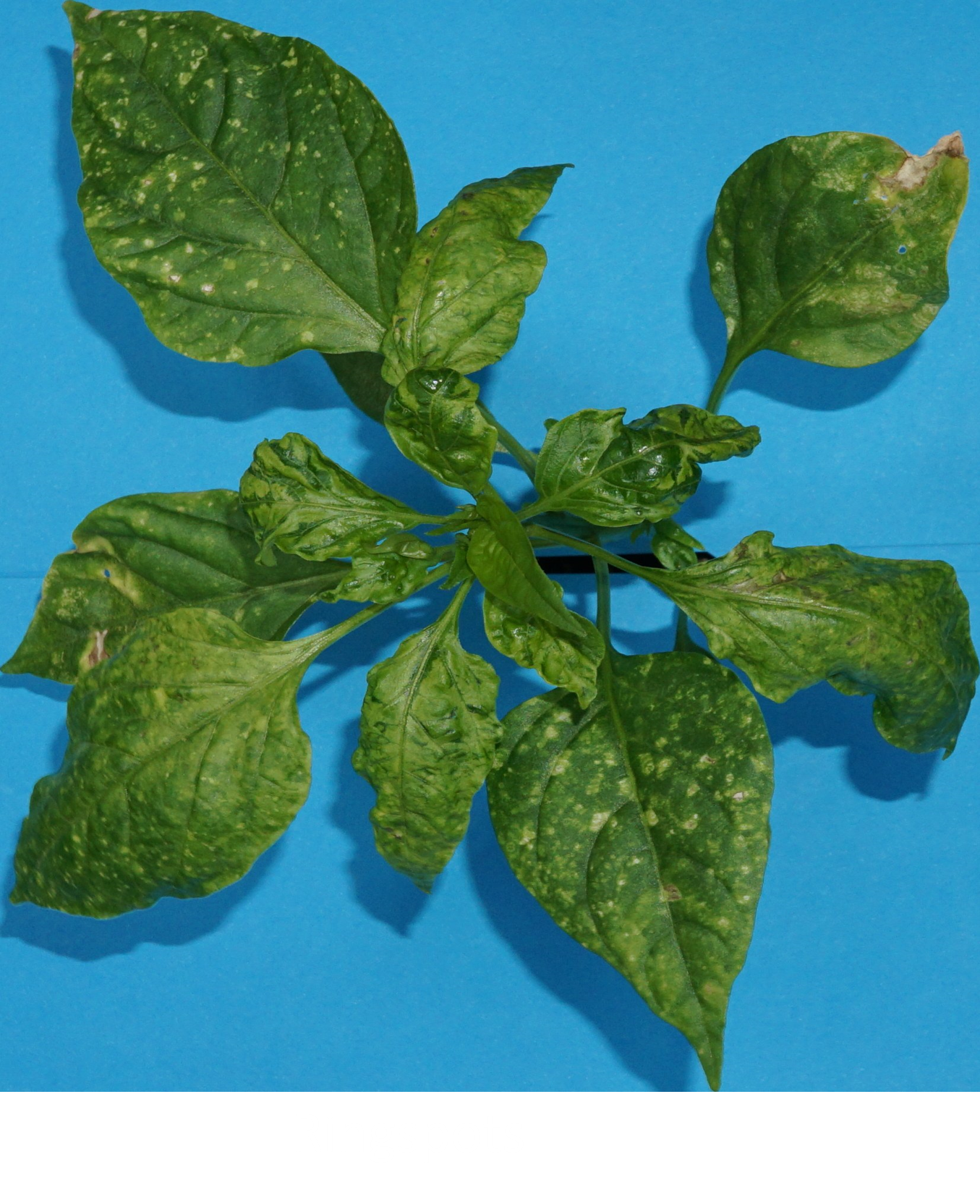

Supplement: Supplementary file 1 [file plants-13-03447-s001.zip › 9. Rings pot.png]
